# Patient-Specific *Bacteroides* Genome Variants in Pouchitis

**DOI:** 10.1128/mBio.01713-16

**Published:** 2016-11-15

**Authors:** Joseph H. Vineis, Daina L. Ringus, Hilary G. Morrison, Tom O. Delmont, Sushila Dalal, Laura H. Raffals, Dionysios A. Antonopoulos, David T. Rubin, A. Murat Eren, Eugene B. Chang, Mitchell L. Sogin

**Affiliations:** a Josephine Bay Paul Center, Marine Biological Laboratory, Woods Hole, Massachusetts, USA; b Section of Gastroenterology, Department of Medicine, Knapp Center for Biomedical Discovery, The University of Chicago, Chicago, Illinois, USA; c Department of Internal Medicine, Division of Gastroenterology and Hepatology, Mayo Clinic, Rochester, Minnesota, USA; d Biosciences Division, Argonne National Laboratory, Argonne, Illinois, USA

## Abstract

A 2-year longitudinal microbiome study of 22 patients who underwent colectomy with an ileal pouch anal anastomosis detected significant increases in distinct populations of *Bacteroides* during 9 of 11 patient visits that coincided with inflammation (pouchitis). Oligotyping and metagenomic short-read annotation identified *Bacteroides* populations that occurred in early samples, bloomed during inflammation, and reappeared after antibiotic treatment. Targeted cultivation of *Bacteroides* isolates from the same individual at multiple time points and from several patients detected subtle genomic changes, including the identification of rapidly evolving genomic elements that differentiate isogenic strains of *Bacteroides fragilis* from the mucosa versus lumen. Each patient harbored *Bacteroides* spp. that are closely related to commonly occurring clinical isolates, including *Bacteroides ovatus*, *B. thetaiotaomicron*, *B. vulgatus*, and *B. fragilis*, which contained unique loci in different patients for synthesis of capsular polysaccharides. The presence of unique *Bacteroides* capsular polysaccharide loci within different hosts and between the lumen and mucosa may represent adaptations to stimulate, suppress, and evade host-specific immune responses at different microsites of the ileal pouch.

## IMPORTANCE

This longitudinal study provides an opportunity to describe shifts in the microbiomes of individual patients who suffer from ulcerative colitis (UC) prior to and following inflammation. Pouchitis serves as a model for UC with a predictable incidence of disease onset and enables prospective longitudinal investigations of UC etiology prior to inflammation. Because of insufficient criteria for predicting which patients will develop UC or pouchitis, the interpretation of cross-sectional study designs suffers from lack of information about the microbiome structure and host gene expression patterns that directly correlate with the onset of disease. Our unique longitudinal study design allows each patient to serve as their own control, providing information about the state of the microbiome and host prior to and during the course of disease. Of significance to the broader community, this study identifies microbial strains that may have genetic elements that trigger the onset of disease in susceptible hosts.

## INTRODUCTION

Cross-sectional studies have described dysbiosis (1, 2) and a large number of host genes and single nucleotide polymorphisms (3, 4) associated with ulcerative colitis (UC), one of the inflammatory bowel diseases (IBD) that cause chronic inflammation of the colon. Because clinicians lack criteria for predicting the onset of UC, cross-sectional studies that compare UC patients with individuals presumed to be healthy cannot unambiguously attribute shifts in microbial communities or altered host gene expression patterns to initial inflammation events. Large interindividual differences in gut microbiota will confound attempts to identify meaningful associations between shifts in the microbial community and onset of disease. In contrast, longitudinal studies of host gene expression and microbiome communities for individual patients prior to and after the onset of UC minimizes the influence of confounding factors that obscure cause-effect relationships.

Patients with medically refractory UC often choose to undergo surgical intervention to achieve cure and continence, which involves a colectomy with an ileal pouch anal anastomosis (IPAA). The ileal pouch functions as a new reservoir to store stool and undergoes physiologic changes to become more “colon-like” within the first 4 months, including colonic epithelial function and a microbial composition similar to that residing in the colon (5, 6). Although the ileal tissue is initially normal, nearly half of the patients develop inflammation of the pouch (pouchitis), which exhibits histologic and endoscopic features similar to UC (7). The similarities between pouchitis and UC coupled with the predictable incidence of pouchitis enables prospective longitudinal investigations of UC etiology prior to inflammation.

Cross-sectional studies of pouchitis patients show that the biopsy site and initial inflammation covary with changes in host transcripts, whereas shifts in the pouch microbial community detected by marker gene analyses correlate only with antibiotic treatment (8). Beyond the inherent limitation of cross-sectional studies that do not include samples from the same patient before and after onset of inflammation, marker gene analyses that focus on rRNA gene targets might lack resolution required for detecting subtle shifts in relative abundance of pathobionts and naturally occurring host-associated microbes with nearly identical genomes. In contrast to large cross-sectional studies, marker gene and shotgun metagenomic analyses in longitudinal studies provide a means to account for pouch microbiome differences between the healthy and inflamed pouch within an individual patient. The assembly of shotgun metagenomic reads into contigs and assembled genomes have the potential to report differences in rapidly evolving genomic regions of closely related organisms. Such differences might represent horizontal gene transfers between *Bacteroides*, including genes that specify capsular polysaccharide (CPS) biosynthesis (9), which can either stimulate or suppress an immune response (10–13), and conjugative transposons that mobilize toxin genes (14).

We used a combination of 16S rRNA marker genes, shotgun metagenomics, cultivation, and assembly-based metagenomics to survey the gut microbial communities over a 2-year period after patients had undergone IPAA. The marker gene and shotgun metagenomic short reads reported changes in the relative abundance of potential pathobionts in response to inflammation and antibiotic stress throughout the course of disease for individual patients. The combination of cultivation, assembly-based metagenomics, and read mapping to cultivar genomes detected subtle genomic changes mediated by putative horizontal gene transfer events before, during, and after inflammation events within the IPAA pouches of individual patients (15).

## RESULTS

### Patient sampling.

**FIG 1:**
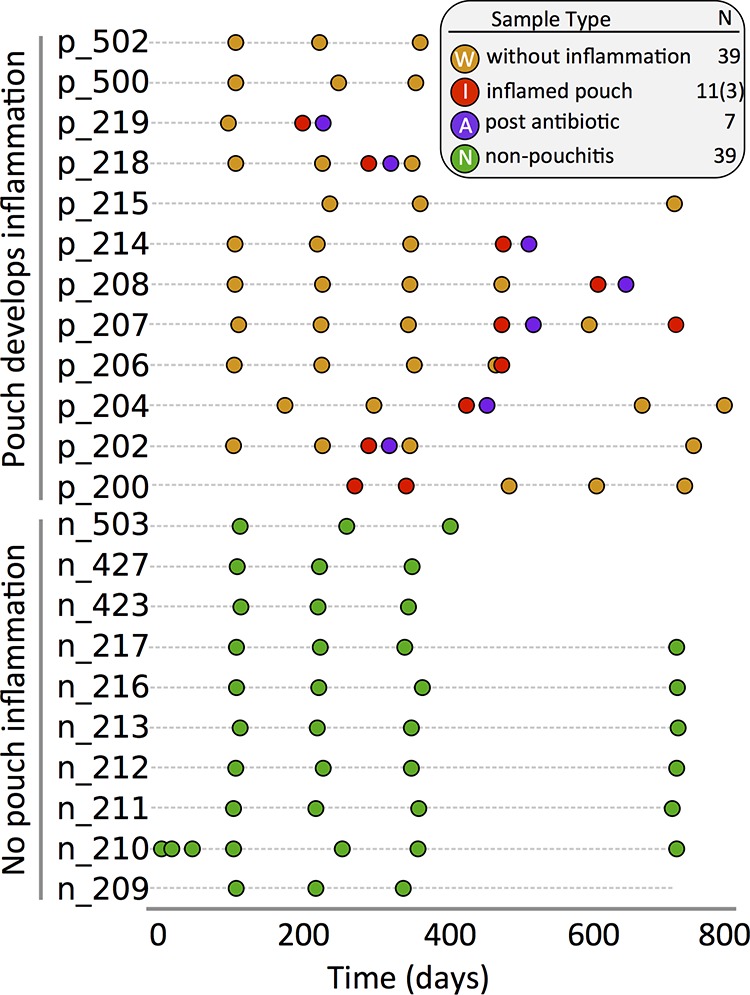
Longitudinal sampling of pouchitis and nonpouchitis patients. Samples from p_patients (patients who experienced inflamed ileal pouches at least once during the 2-year study) were collected during periods of inflammation (inflamed pouch), in the absence of inflammation, and 30 days after administration of antibiotics (post antibiotic). Samples were also collected from n_patients who never developed inflammation during the study period (non-pouchitis).

Based upon the pouch disease activity index, 9 patients developed pouchitis at least once during the 2-year study period (p_patients), 10 patients never developed pouch inflammation (n_patients), and 3 patients (p-500, p-502, and p-215) developed pouchitis after completion of the study (see Table S1 in the supplemental material). This 2-year longitudinal study included 96 luminal content samples acquired at different time points after functionalization of the ileal pouch (Fig. 1) from IBD patients during visits where they exhibited inflammation (inflamed visits) (I samples), during visits where they did not exhibit inflammation (visits without inflammation) (W samples), and approximately 30 days following antibiotic treatment prescribed for pouchitis (A samples). Thirty-nine of the luminal samples came from surgically created pouches that never became inflamed throughout the duration of the study (N samples), and a brush sampling procedure recovered microbes acquired from three mucosal samples (GG sampling site) during periods of inflammation after pouch functionalization.

### Marker gene analysis.

Amplicon sequencing from patient samples recovered an average of 57,682 v4v5 rRNA marker gene sequences from each of 92 samples. Sequences that GAST (Global Alignment for Sequence Taxonomy) analyses (16) resolved to *Bacteroides* ranged from 20 to 96% relative abundance during 8 of 11 periods of inflammation (see Fig. S1 and S2 in the supplemental material). Other genera and families (taxa) that commonly occur at relatively high abundance in the human gut, e.g., *Clostridium*, *Enterobacteriaceae*, and *Streptococcus*, represented relatively low-abundance taxa in samples from inflamed visits. The linear mixed-effects models (LMMs) estimated an average increase in the relative abundance of *Bacteroides* of 27% (97.5% confidence interval [97.5% CI], 10.4% to 43.4%) during inflammation versus all other samples (Benjamini-Hochberg [BH]-adjusted *P* value of 0.073). The *Cetobacterium* model estimate of 4.1% (97.5% CI, −0.057% to 14.1%) increase during inflammation was the second highest among all taxa. The top three taxa that negatively correlated with inflammation included *Lachnospiraceae*, *Streptococcus*, and *Enterobacteriaceae* with percent decrease of 8.4, 7.9, and 6.2, respectively (Table S2). Microbial communities significantly differed (i) among patients (all samples from one patient versus all samples from another patient), (ii) between p_patients and n_patients, and (iii) among pouch conditions at the time of sampling, and these three differences account for 43%, 3%, and 3% of the total variation, respectively (Table 1). The analysis of variance (ANOVA) of the relative abundance of *Bacteroides* reported significant differences between the W and I samples (*P* = 0.02) (Table 3; Fig. S2) but no significant differences in pairwise comparisons of samples from all other states.

**TABLE 1:** ADONIS and dispersion tests for differences in community composition

Comparison	MED			GAST			MG-RAST		
	*R*^2^	*P* value	Disp^d^	*R*^2^	*P* value	Disp	*R*^2^	*P* value	Disp
Patient group^a^	0.02	<0.001	0.105	0.03	<0.001	0.091	0.01	0.169	0.159
Condition^b^	0.02	0.001	0.762	0.03	<0.001	0.327	0.03	0.037	0.12
Patient^c^	0.49	<0.001	<0.001	0.43	<0.001	<0.001	0.30	<0.001	<0.001

a Comparison between samples from pouchitis and nonpouchitis patients.

b Comparison among samples taken from a pouch after antibiotic treatment, during inflammation, uninflamed pouchitis patient, and non-pouchitis patients.

c Comparison among patients.

d *P* value for the test of equal variation (dispersion) among groups (null hypothesis of no difference).

**FIG 2:**
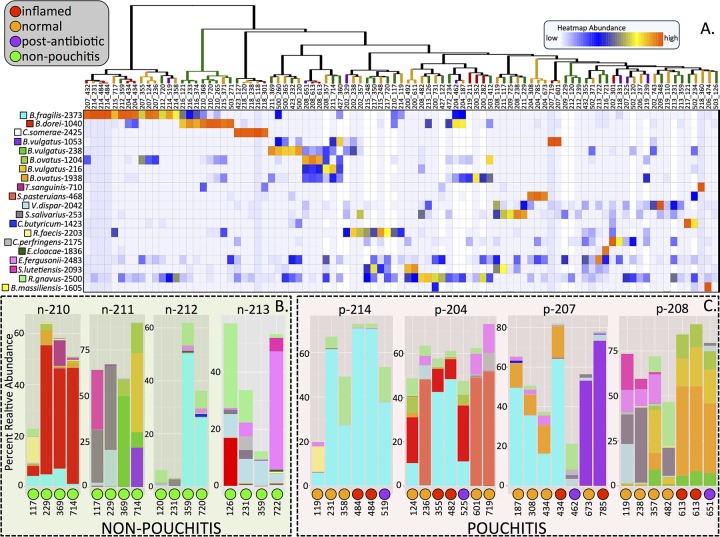
(A) Cluster dendrogram and heat map for the 20 most abundant oligotypes. Clustering employed average linkage of a Bray-Curtis dissimilarity matrix. The labels for terminal branches show the patient number first (preceded by “p” or “n” indicating whether the patient eventually develops pouchitis elsewhere) and then the number of days after initialization of the pouch; the colors of the terminal branches indicate the condition of the pouch at the time of sampling. The heat map represents scaled value for relative abundance of each oligotype per sample. The labels to the left of the heat map indicate the best match of the oligotype sequence in the NCBI RefSeq RNA database followed by the unique oligotype identifier (ID). Longitudinal patterns of nonpouchitis (B) and pouchitis (C) patients are displayed as bar plots. The patient ID is shown above each individual bar plot. The color of the bar corresponds with the oligotype ID of the heat map. The condition of the pouch and the number of days elapsed since pouch activation are displayed below each bar plot.

Minimum entropy decomposition (MED) analysis, which employs Shannon entropy calculations to differentiate marker gene sequences that may differ by only a single nucleotide (17), resolved 312 distinct oligotypes (18) and described beta diversity that according to permutational multivariate analysis of variance (PERMANOVA) tests are nearly identical to the analysis of GAST genus-level taxon assignments. Community composition (Fig. 2) significantly differed among patients, among N, I, W, and A samples and between p_patients and n_patients (Table 1). A significant shift in microbial communities of pouchitis patients involved expansion of *Bacteroides* from a mean relative abundance of 20% prior to inflammation to 50% in I samples (see Fig. S2 in the supplemental material). For the entire pouchitis patient cohort, oligotypes identified rRNA sequences with perfect matches to the NCBI RefSeq RNA database for *Bacteroides fragilis* (1 oligotype), *B. ovatus* (2 oligotypes) and *B. vulgatus* (3 oligotypes) during 9 of the 11 periods of inflammation (Fig. 2) with a minimum, maximum, and average percent relative abundance of 0.44, 73, and 35.7, respectively (Table S3). A single *B. fragilis* oligotype (2373) represented the dominant organism in 3 of the 11 samples taken during inflammation (Fig. 2), and LMMs predicted the highest increase in relative abundance of any oligotype (10.9%; 97.5% CI, 2.1 to 19.9) (BH-adjusted *P* value of 0.232) during inflammation (Table S2). Some patients exhibited increases for more than one oligotype that resolved to the same species. For example, the p-208 I sample contained abundant *B. ovatus* and *B. vulgatus*, each represented by two distinct oligotypes (Fig. 2). LMMs predicted positive inflammation coefficients for several *B. vulgatus* and *B. ovatus* oligotypes (Table S2). In three patients (p-213, p-204, and p-207), the *B. fragilis* oligotype 2373 represented the most abundant population in the microbiome during inflammation and was present more than 300 days prior to the onset of inflammation. The same oligotype occurred in relatively high abundance in patients who did not develop inflammation (p-212 and p-210). LMMs predicted positive correlation between several low-abundance taxa with inflammation. Oligotype 1114, with exact sequence similarity to *B. faecis* and *B. thetaiotaomicron*, increased, on average, 2.3% during inflammation. LMMs estimated negative correlations between inflammation and oligotypes with high sequence similarity to *Streptococcus* and *Lachnospiraceae*, but decreases never exceeded 3.5% (Table S2).

### Metagenomic short-read functional potential and taxonomy.

Shotgun metagenomic sequencing, quality filtering, and merging of paired-end reads from 170-bp insert libraries for 99 samples (96 luminal samples plus 3 mucosal samples) yielded 555 Gb (~33 million reads/sample). MG-RAST (metagenomic rapid annotation of microbial genomes using subsystems technology) (19) annotation of all samples identified 13,325 functions. However, the functional potential of microbial communities did not differ between n_patients and p_patients or among W, I, A, and N samples (see Fig. S3 in the supplemental material). Functional potential differed significantly among patients and explained 29% of the variation (*P* = 0.001). In agreement with the 16S rRNA estimates, the mean relative abundance of *Bacteroides*, according to taxonomic assignment of short reads to the M5nr (nr stands for nonredundant) protein database, differed significantly in I samples compared to W samples (Table 3; Fig. S2).

### Assembly and temporal changes in metagenome assembled genomes.

**FIG 3:**
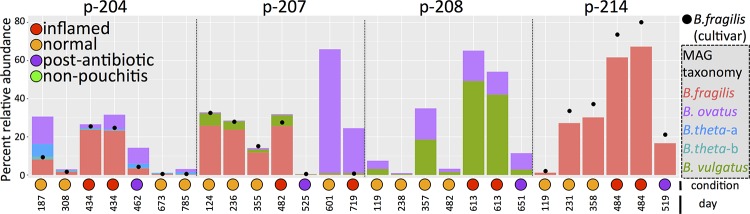
Bar plots show the temporal change in percent abundance of *B. fragilis*, *B. vulgatus*, *B. ovatus*, and *B. thetaiotaomicron* MAGs recovered from four patients that developed pouchitis. The letters “a” and “b” in *B. theta*-a and *B. theta*-b denote two different MAGs that match *B. thetaiotaomicron*. The condition of the pouch during each sampling is indicated by the color of the circle below each bar. Each MAG derived from the sample is displayed in chronological order as the number of days following pouch activation. The relative abundance of each *B. fragilis* cultivar isolated from patients p204, p207, and p214 is displayed as a black dot within the plot.

Each metagenome assembly included reads from all longitudinal samples for each patient. Using assembled contigs of >5 kbp, anvi’o (20) displayed 63 *Bacteroides* “metagenome assembled genomes” (MAGs) with an average size, completion, and redundancy of 4.18 Mbp, 58.8%, and 4.6%, respectively. These MAGs describe 12 *B. fragilis*, 4 *B. ovatus*, 12 *B. thetaiotaomicron*, and 11 *B. vulgatus* draft genomes. One or more of these genomes were present in 9 of the 11 samples collected during inflammation (Fig. 3), but for any one patient, only a single *B. fragilis* MAG assembled in the metagenome of pooled luminal (M) samples. Based upon mapping short reads back to the assembly, the abundance of the *Bacteroides* group (*B. fragilis*, *B. ovatus*, *B. vulgatus*, and *B. thetaiotaomicron*) was significantly greater in I samples than in N and W samples (Table 3; see also Fig. S2 in the supplemental material). We isolated an additional 354 MAGs longer than 1.5 Mbp with no more than 10% single-copy gene redundancy (Table S4).

### Functional analyses of MAGs.

Annotation of the 63 *Bacteroides* MAGs produced a set of 7,011 distinct functions. Each genome contained an average of 3,468 genes, and the identified functions of *Bacteroides* MAGs did not differ significantly between n_patients and p_patients (Table 2; see also Fig. S4 in the supplemental material). The species-level classification explained 54% of the variation in functional assignments with MAGs that form tight clusters differing at multiple loci. For example, the *B. fragilis* p207-33 (patient 207 sample taken 33 days after pouch activation) and *B. fragilis* p212-31 MAGs are 93% and 97% complete, respectively (Table S4), and each contains ~190 unique gene features.

**TABLE 2:** Functional diversity of *Bacteroides* MAGs^a^

Comparison	*R*^2^	*P* value	Disp^e^
Patient group^b^	0.01	0.87	0.607
Species^c^	0.54	<0.001	<0.001
Patient^d^	0.21	1	<0.001

a Summary of the results of ADONIS and dispersion tests for *Bacteroides* functions. We tested whether there were differences in the functions of all *Bacteroides* MAGs in the p_patient versus n_patient groups, whether there were significant differences in the functional potential of species, and whether patients harbor *Bacteroides* with a distinct functional potential.

b Comparison between samples from pouchitis and nonpouchitis patients.

c Comparison of functional potential based on RAST species assignment.

d Comparison among patients.

e *P* value for the test of equal variation (dispersion) among groups (null hypothesis of no difference).

**TABLE 3:** ANOVA of *Bacteroides* relative abundance^a^

Comparison	GAST		MG-RAST		MAGs	
	Diff	*P* value	Diff	*P* value	Diff	*P* value
I vs A	27.88	0.17	26.51	0.19	18.86	0.07
N vs A	3.64	0.99	5.25	0.97	−4.43	0.91
W vs A	−2.09	1.00	−2.64	1.00	5.25	0.86
N vs I	−24.24	0.06	−21.26	0.08	−23.29	0.00
W vs I	−29.97	0.02	−29.15	0.01	−13.61	0.05
W vs N	−5.73	0.80	−7.89	0.63	9.68	0.05

a The results of the ANOVA and Tukey’s HSD test among inflamed (I), antibiotic (A), without pouchitis (W), and n_patient (N) samples are summarized for three different estimates of *Bacteroides* relative abundance (GAST, MG-RAST, and MAGs). The ANOVA and Tukey's HSD test are also reported for differences in the percentage of metagenomic read recruitment to three *Bacteroide fragilis* cultivars isolated from two pouchitis patients (p-207 and p-214) and one nonpouchitis patient (n-216). The groups include metagenomic samples from the HMPDACC and I, A, W, and N samples. Diff indicates the difference in percent relative abundance between the two groups being compared. The *P* values of the ANOVA for the three estimates of abundance are as follows: *P* = 0.281 for GAST, *P* = 0.017 for MG-RAST, and *P* < 0.001 for MAGS. The *P* values for the ANOVA of *B. fragilis* cultivar relative abundance were all <0.001.

The high relative abundance of clinically relevant *Bacteroides* during inflammation motivated an investigation of the genes within rapidly evolving chromosomal regions. We initially focused our attention on gene clusters necessary for capsular polysaccharide (CPS) production. These functionally related but compositionally distinct gene clusters can occur multiple times within highly variable regions of the *Bacteroides* genome (9). The composition of genes necessary for CPS biosynthesis, including transcriptional regulatory elements (UpxY and UpxZ), coding regions for glycosyltransferase genes, UDP-*N*-acetylglucosamine 2-epimerase (EC 5.1.3.14), and glucosamine-6-phosphate deaminase (EC 3.5.99.6) did not significantly differ among patients or between pouchitis and nonpouchitis patients. The correlation between the assemblage of CPS loci and RAST species assignment was high (*R*^2^ = 0.44) and significant (*P* = 0.001). The *B. fragilis*, *B. thetaiotaomicron*, *B. ovatus*, and *B. stercoris* MAGs contain a mean of 64, 52, 45, and 38 CPS biosynthetic cluster genes, respectively, while *B. vulgatus* contained a mean of 35 such genes per genome. Each of the *B. fragilis* genomes contained multiple copies of the *upxY* and *upxZ* loci responsible for transcriptional regulation of CPS. The number of *upxZ* regulatory loci in *B. fragilis* MAGs ranged from two to eight distinct copies. The MAG that contained only two regulatory element coding regions assembled to ~50% of the length of other *B. fragilis* genomes and was isolated from an n_patient. The MAGs of *B. thetaiotaomicron* but not *B. ovatus* also contained these regulatory genes*.* The composition of the genes downstream of the regulatory elements in *B. fragilis* varied in gene number and content within and between each of the genomes and included several genes that are known to be virulent within other genera (see Fig. S4 in the supplemental material). We detected genes necessary to metabolize sialic acids, including *N-*acetylneuraminic acid (Neu5Ac) including *neuB* (*N*-acetylneuraminate synthase [EC 2.5.1.56]) and *mnaA* (UDP-*N*-acetylglucosamine 2-epimerase [EC 5.1.3.14]) in each of the *B. fragilis* MAGs isolated from pouchitis patients. Virulence factors in the *Bacteroides* MAGs recovered at high abundance from inflamed samples included colicin V production (colicin V [PF02674]), bile hydrolysis (choloylglycine hydrolase [EC 3.5.1.24]), resistance to mercury (mercuric ion reductase [EC 1.16.1.1]), resistance to copper (CutC [PF03932]), and a mycobacterium-like virulence operon possibly involved in quinolinate biosynthesis (quinolinate synthetase [EC 2.5.1.72], l-aspartate oxidase [EC 1.4.3.16], and quinolinate phosphoribosyltransferase [EC 2.3.2.19]). None of the MAGs contained contigs with sequence similarity to the *Bacteroides fragilis* toxin (BFT) according to an hidden Markov model (HMM) search for the N-terminal domain of fragilysin (PF16376).

### *Bacteroides* cultivar genomes.

Cultivation efforts yielded 14 *Bacteroides* isolates from I, W, and N samples from four p_patients and two n_patients. The isolation of cultivars and determination of their genome sequence confirmed draft genome assemblies from the shotgun metagenome and described differences between related genomes. In this study, each of the 14 *Bacteroides* cultivar shotgun genomic data sets assembled into draft genomes with a minimum length of 4.4 Mbp and contained more than 4,217 coding sequences and 354 to 356 RAST (21) subsystems. The genomes of cultivars isolated from patients p-212, p-214, p-207, p-215, and n-216 represent distinct bacterial isolates that share strong homology with *B. fragilis* in the RAST database. The p-219 cultivar genome resolves to *B. ovatus*. Reciprocal mapping of short reads from each *B. fragilis* cultivar assembly to all other assembled cultivar genome sequences in this study demonstrates that each short read is derived from a distinct but very closely related *B. fragilis* MAG with the same oligotype. In agreement with observations of only one *B. fragilis* MAG for pooled longitudinal samples for a given patient, each sequenced *B. fragilis* cultivar genome occurred in only a single patient. MAUVE alignment (22) of the cultivar and corresponding MAG contained an average of 94% shared nucleotide identity, and the correlation between the relative abundance of metagenomic short reads recruited by the MAG and cultivar was high and significant (*R*^2^ = 0.996; *P* value of <0.0001). Unaligned regions of the cultivar genome contained 45 to 332 genes (1 to 7% of the genome) that were missing in MAGs isolated from the same patient. Genes that define transposons, ribosomal proteins, and hypothetical proteins accounted for 69 to 97% of these coding region differences.

### Metagenomic and reciprocal cultivar mapping.

**FIG 4:**
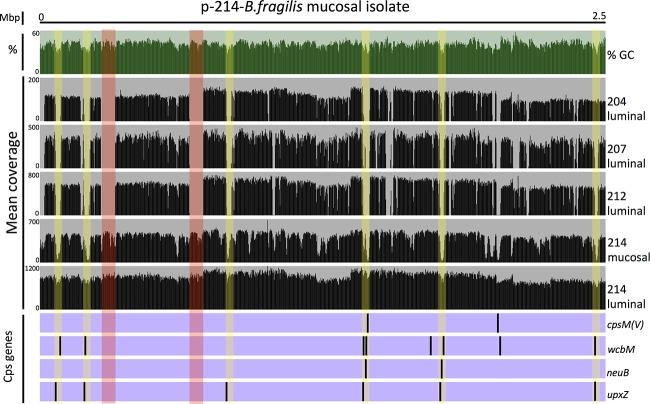
Read mapping to the p-214 *B. fragilis* genome cultivar isolated during inflammation. The top green histogram shows the percent GC for 2-kbp segments over 2.5 Mbp of the 5-Mbp draft genome from the *B. fragilis* cultivar isolated from the inflamed pouch of patient 214. The black histograms in the middle of the figure represent mean coverage over 2-kbp genomic segments of short metagenomic reads (luminal or mucosal samples collected during inflammation from patients p-204, p-207, p-214, and from the lumen of the nonpouchitis patient n-212) that mapped 100% over their length with 97% identity to the p-214 cultivar genome. Black bars on the purple background at the bottom of the figure display the positions of four genes [*cpsM*(*V*), *wcbM*, *neuB*, and *upxZ*] within capsular polysaccharide biosynthesis (CPS) loci highlighted in yellow. Two regions highlighted in red represent mobile elements within the genome.

Mapping short reads from each metagenomic data set to the assembled *Bacteroides* cultivar genomes enables assessments of genome coverage (estimated by read recruitment) and detection of potential genome insertions, deletions, and successions (by new populations) in longitudinal studies of each patient. The short metagenome reads for longitudinal luminal samples from patient 204 cover 100% of the p-204 *B. fragilis* cultivar genome sequence (see Fig. S5 in the supplemental material). In contrast, patterns of read recruitment to the p-214 cultivar genome differed for short metagenomic sequences for longitudinal luminal samples collected from the nonpouchitis patient n-212 and from pouchitis patients p-204 and p-207 (Fig. 4). These data indicate that each patient harbors a unique *B. fragilis* that remains stable over time. Many regions from different patient metagenome samples that display low or no read coverage when mapped to the p-214 genome encode proteins that function in CPS biosynthesis, including CpsM, WcbM, and NeuB, while other examples indicate a clustering of genes that likely play a role in transposition. Read mapping to the p-214 cultivar also revealed differences between closely related *B. fragilis* in the luminal versus mucosal samples. The high-coverage regions represent conserved genetic elements shared between *B. fragilis* genomes from the lumen and mucosa, whereas the low-coverage regions represent putative differences between genomes of the dominant *B. fragilis* populations of the lumen and mucosa (Fig. 4). Failure to map reads to the cultivar genome sequence provides compelling evidence that *B. fragilis* in the luminal samples of patient p-214 differs not only from the *B. fragilis* in the mucosal sample of patient p-214 but also differs in the same regions from *B. fragilis* in the other pouchitis and nonpouchitis patients in this study. This variability in *B. fragilis* genomes also appears in mapping experiments where the p-214 *B. fragilis* cultivar genome recruited very few reads from metagenomes that represent 154 healthy samples in the Human Microbiome Project Data Analysis and Coordination Center (http://hmpdacc.org) (Table 4; see also Fig. S6 at doi:10.6084/m9.figshare.3851481). The mean relative abundance of *B. fragilis* in the Human Microbiome Project (HMP) samples was 1.5% with a maximum of 19.7%, which compares to an average of 5.8% with a maximum of 79.5% in this study. Several regions of the genome receiving significant coverage contain several conjugative transposons, antibiotic resistance genes, and toxin-antitoxin genes (Table S5; see also Fig. S6 at doi:10.6084/m9.figshare.3851481).

**TABLE 4:** ANOVA of *B. fragilis* read recruitment^a^

Comparison	p-214		p-207		n-216	
	Diff	*P* value	Diff	*P* value	Diff	*P* value
A vs HMPDACC	3.89	0.75	2.98	0.83	3.00	0.83
I vs HMPDACC	12.88	0.00	11.35	0.00	11.36	0.00
N vs HMPDACC	1.52	0.86	1.88	0.63	1.85	0.66
W vs HMPDACC	3.09	0.27	3.36	0.10	3.09	0.16
I vs A	8.99	0.14	8.37	0.10	8.36	0.11
N vs A	−2.37	0.96	−1.10	1.00	−1.16	1.00
W vs A	−0.80	1.00	0.38	1.00	0.08	1.00
N vs I	−11.37	0.00	−9.47	0.00	−9.52	0.00
W vs I	−9.80	0.00	−7.99	0.01	−8.27	0.00
W vs N	1.57	0.93	1.48	0.91	1.24	0.95

a See Table 3 footnote for details.

## DISCUSSION

Close examination of several *B. fragilis* cultivars isolated from longitudinal luminal samples combined with metagenomic read mapping demonstrated that each patient harbored a distinct *B. fragilis* population. These potential pathobionts were present early after the creation of the pouch and persisted for at least 2 years, remaining at relatively high abundance during periods of health, inflammation, and postantibiotic treatment. Comparisons of luminal and mucosal samples from inflamed visits of the same patient detected two distinct *B. fragilis* populations, one of which could be detected only in mucosal samples. Comparisons of genomes from these organisms revealed that their differences principally occurred within multiple capsular polysaccharide biosynthesis loci that contribute to microcapsule production and typically contain transcriptional regulatory elements (UpxY and UpxZ), coding regions for glycosyltransferase genes, UDP-*N*-acetylglucosamine 2-epimerase (EC 5.1.3.14), and glucosamine-6-phosphate deaminase (EC 3.5.99.6). While *Bacteroides* organisms are abundant members of the human intestinal tract, the relatively abundant species during inflammation in this study (*B. fragilis*, *B. ovatus*, *B. vulgatus*, and *B. thetaiotaomicron*) also commonly occur in association with anaerobic infection and IBD (23–25).

These *Bacteroides* represent known opportunistic pathogens that can reach relative abundance of >80% in IBD patients with cell numbers exceeding 10^9^ cell/ml at the mucosal layer (25, 26). The ability of *Bacteroides* to maintain high abundance during pouch inflammation and normal epithelial function in the pouch likely reflects their ability to activate, suppress, or evade an overly active immune response during W, I, and A sampling visits, respectively. To maintain the abundance patterns observed in this study, members of the genus must exploit a mechanism to survive within each of these unique host environments. Several *Bacteroides* species (*B*. *thetaiotaomicron*, *B. ovatus*, *B. fragilis*, and *B. vulgatus*) have evolved multiple distinct capsular polysaccharides through close interaction with host cells and other microbes within the human intestinal tract. *Bacteroides* have evolved diverse capsules that include both lipopolysaccharide and polysaccharide (25). *B. fragilis* in this study contains at least eight unique CPS biosynthesis loci that correspond to zwitterionic polysaccharide A to polysaccharide H (PSA-PSH), which serve an essential role in the growth, virulence, and adaptation to variable conditions (27). The inversion of the promoter region regulates each polysaccharide loci (28), but the UpxY and UpxZ proteins dynamically control expression (29). The expression of a single CPS operon and location of its polysaccharide product on the surface in any given cell lead to a population of antigenically diverse cells (30). This population-level variability facilitates the survival of *B. fragilis* in many environments and its ability to maintain significant relative abundance without causing inflammation. Although the flanking regions of the PSA-PSH are largely conserved, our mapping results indicate little to no homology between the PSA-PSH contained in the well-characterized *B. fragilis* NCTC 9343 or among each of the three cultivars identified in this study. These results agree with other characterizations of *B. fragilis* CPS diversity (9). Figure 4 reveals that the percent GC content of CPS loci differs from the surrounding genome, consistent with recent transfer events and/or recombination within the genome (31, 32).

Some of the CPS loci in *Bacteroides* MAGs contain genes (e.g., *neuB*, *wcbM*, and *cpsM*) that define virulence factors in other pathogens. For example, the protein product of *neuB* catalyzes the final step in the biosynthesis of the sialic acid Neu5Ac (33). The potential decoration of CPS with sialic acids in *Bacteroides* cultivars from p_patients resembles mechanisms for avoiding or causing host innate immune response in other virulent organisms, e.g., *Campylobacter jejuni*, *Escherichia coli*, *Neisseria meningitidis*, and *Streptococcus suis* (34). Several *B. fragilis* CPS loci in this study contain genes encoding other elements that also occur within the capsules of known virulent genomes, including *cpsM* from the capsule of *Streptococcus pneumoniae* and *Burkholderia pseudomallei* wcbM (35), which shares strong homology with *hddC* from *C. jejuni* (36). The effect of *wcbM*, *cpsM*, and *neuB* in *B. fragilis* on virulence remains unclear, but their presence within the genome of other *Bacteroides* in this study suggests that they may contribute to the cause and/or elevation of a host immune response.

Previous reports have reported horizontal gene transfer between *Bacteroides* through pairwise comparison of genome sequences from isolates (37, 38). Comparisons of the MAGs and cultivar genomes in this study provide suggestive evidence of gene transfer between *Bacteroides*. For example, both a *B. fragilis* MAG and the same genome from a cultivar draft genome from p-207 shared nearly 100% sequence identity with several genomic regions of a MAG for *B. vulgatus*. The shared sequences spanned coding regions for several outer membrane receptors, site-specific recombinase, and integrative conjugative elements (ICEs) containing tetracycline resistance genes. The presence of these genes within independent blooms of two different species during inflammation suggests that they provide an adaptive advantage. We also detected genes shared among some *Bacteroides* from all sample types for multiple patients, including ICEs, membrane-related proteins, transcriptional regulatory genes, mobile element proteins, outer membrane receptor proteins, and multidrug resistance genes that confer increased fitness to *Bacteroides*. ICEs in particular can be modified by transposons, resulting in the insertion of genes from the recipient genome. This process can yield a customized ICE that benefits subsequent *Bacteroides* colonization of the gut (37). They exhibited high copy numbers (10 copies) in relation to the surrounding genome of conjugative transposons (CTn) and related genes common to CTn-like ICEs. The high copy numbers indicate that these elements excise from the genome and replicate prior to moving to a recipient genome within the pouch, consistent with previous work (39). Because transfer events increase within inflamed environments, movement of these genes in the pouch environment may be elevated among *Bacteroides* and has the potential to transfer virulence genes from the CPS of *B. fragilis* to other species and possibly across genera.

While antibiotics seemed to reduce the severity of disease (1, 2, 8), the abundance of *Bacteroides* did not significantly decrease, and in three patients, the same *Bacteroides* returned 1 month after antibiotic treatment was completed. The reestablishment of *Bacteroides* following antibiotic treatment could reflect the presence of multiple antibiotic resistance genes (40), which spread by conjugation and mobilization (34). Alternatively, the same *Bacteroides* might reestablish from external sources or from survivors of the original antibiotic treatment. Although *B. fragilis* was undetected following antibiotic treatment in p-207, a previously undetected *B. vulgatus* became dominant during refractory inflammation with functional similarity to a *B. vulgatus* from patient p-208 and contained ICEs carrying genes necessary for tetracycline and clindamycin resistance. Ciprofloxacin was used to treat pouchitis in this study, and it is unknown whether tetracycline or clindamycin genes would confer resistance to this antibiotic.

The high coverage of marker gene analysis contributes to reduced statistical variability when profiling microbial communities and enables the detection of rare taxa. Yet, the reliance upon a single locus constrains the level of information for resolving differences between closely related taxa. MED’s ability to resolve amplicon sequences into oligotypes that differ by a single nucleotide (11) offers increased resolution relative to taxonomy and clustering techniques (41, 42), but neither MED nor the MG-RAST annotation of short metagenomic reads detected community shifts revealed through comparisons of the MAGs. Despite having identical oligotypes and essentially identical functional annotations, read mapping to cultivar draft genomes described differences between *B. fragilis* populations from different patients and between luminal samples (M sample type) versus mucosal samples (GG sample type) taken during that same inflammation visit. When combined with longitudinal sampling of a patient, it became possible to track temporal changes in the abundance of closely related but distinct *Bacteroides* populations. Abundant *Bacteroides* species during inflammation were limited to *B. ovatus*, *B. vulgatus*, and *B. fragilis*. Each of these *Bacteroides* genomes was 100% covered over the length of all contigs at least once prior to inflammation. These mapping results provide evidence for the presence of those genomes in the patients’ microbiomes prior to the development of mucosal inflammation. In the case of p-214, the *B. fragilis* genome appeared at least 1 year before inflammation, suggesting that inflammation results from a well-established but low-abundance microbe. Read mapping from individual patient visits revealed significant variation in response to antibiotics among *Bacteroides* genomes. Several of the *Bacteroides* reached 10% relative abundance following antibiotic treatment. In other patients, read mapping did not detect the abundant *Bacteroides* after antibiotic treatment. In the case of p-207, read mapping demonstrated that after antibiotic treatment, *B. vulgatus* replaced the *B. fragilis* that dominated during inflammation. *B. vulgatus* represented 23% of the p-207 microbiome at a second inflammation visit.

Large-scale 16S rRNA surveys that have included hundreds of IBD patients and healthy patient controls have contributed important information about dysbiosis variability but have not yet identified at the genome level specific microbes that could direct diagnosis or treatment of the disease. Although the number of samples in this study is small compared to other studies (8, 43, 44), the longitudinal sampling and clinical data paired with cultivation, shotgun metagenomic assemblies and 16S amplicon sequencing provide unique insights into microbial influences on the development and course of pouchitis. We found a significant difference in the overall community structure between the p_patients and n_patients; however, 3% differences in overall community variation did not provide a useful clinical predictor of inflammation. The relative abundance of *Bacteroides* increased significantly during inflammation, but differences in its relative abundance in p-patients versus n-patients were not significant. The absence of data related to the state of the immune system and other confounding factors may have compromised larger studies where *Bacteroides* either decreased or weakly correlated with IBD (43, 44). The lack of significant decreases in the relative abundance of *Bacteroides* in the antibiotic-treated group highlights the ability of *Bacteroides* to resist certain antibiotics (40, 45). The level of interpersonal variation in overall community structure and the diversity of *Bacteroides* are also important observations. The *Bacteroides* oligotypes were largely stable for a particular individual and occurred across multiple individuals, suggesting that the same *B. fragilis* occurred in multiple patients, yet the *B. fragilis* MAGs that correlate with those operational taxonomic units differed significantly from each other at the nucleotide and functional levels. Similar results for *B. vulgatus* provide further evidence for the lack of sensitivity of 16S rRNA surveys to identify the particular microbial organisms that may be related to the disease.

Despite the abundance of *Bacteroides* and the presence of known virulence genes, low-abundance members of the community might elicit an inflammatory or synergistic interaction between multiple microbes and drive inflammation (12). Two unrelated taxa that were dominant during this study, *Cetobacterium somerae* and *Clostridium perfringens* (44, 45), each might contain genes that contribute to evading an immune response and/or eliciting an abnormal immune response. Different genera and/or variants within the same microbial species likely contribute to the etiology of IBD. Within closely related *Bacteroides*, the patient-specific occurrence of dissimilar genome elements that define functionally related gene clusters have the potential to drive microbial community dynamics and inflammation.

Complex host-microbe relationships leading to pouchitis vary for individual patients. The biological variation may explain why it has been so difficult to find a consistent pathogen in IBD using conventional tools and cross-sectional study designs. Our results underscore the importance of performing prospective studies with highly curated clinical metadata and the application of multiple tools, including high-resolution genomics. The identification of five potentially virulent *Bacteroides* spp. provides specific targets for understanding the underlying mechanisms that lead to refractory pouchitis and potentially IBD. While these conclusions rely principally on abundance patterns from a small group of samples, the known virulence, unique capsule, and correlation with disease index strongly support a role for these species as potential causative agents of disease.

## MATERIALS AND METHODS

### Patient clinical history.

Twenty-two patients recruited at the University of Chicago Medical Center had a previously confirmed diagnosis of ulcerative colitis (UC) and underwent total proctocolectomy with ileal pouch anal anastomosis (IPAA) as standard of care. Each patient underwent endoscopic evaluation of the ileal pouch (pouchoscopy) for collection of luminal contents and mucosal brushings in the ileal pouch. Patients were sampled without bowel lavage or preparation 2 weeks, 4 weeks, 8 weeks, and then every 4 months after pouch functionalization for a period of 2 years.

### Marker gene analysis of pouchitis and nonpouchitis patients.

The marker gene analyses sequenced the v4v5 regions of bacterial 16S rRNA coding regions according to the methods described in reference 46 for each of the 97 samples. A modification of Eren et al. (47) removed low-quality sequences, and GAST (16) assigned taxonomy prior to uploading sequences to the Visualization and Analysis of Microbial Population Structures (VAMPS) website (https://vamps.mbl.edu). Minimum entropy decomposition (MED) identified high-resolution oligotypes (17, 18).

### *Bacteroides* cultivar isolation.

From four p_patients (p-207, p-214, p-215, and p-219) and two n_patients (n-216 and n-212), we isolated 14 *Bacteroides* cultivars from mucosal (GG sample type) and luminal samples (M sample type) from patients at visits without inflammation (W visits) or with inflammation (I) or visits of patients whose surgically created pouches never became inflamed throughout the duration of the study (N visits). Aliquots (50 µl) of patient samples (luminal aspirate or mucosal samples, stored at −80°C) were streaked onto *Bacteroides* bile esculin (BBE) agar and incubated at 37°C, anaerobically, for 72 h. Single colonies substreaked at least twice onto BBE provided an inoculum for overnight anaerobic incubation at 37°C in 10 ml of supplemented brain heart infusion broth. DNA extraction from overnight cultures collected by centrifugation (5,800 × *g*, 10 min, 4°C) followed the DNeasy blood and tissue kit (Qiagen) manufacturer’s instructions for Gram-negative bacteria. Capillary sequencing of PCR amplicons using the universal primers 27F (F stands for forward) and 1525R (R stands for reverse) (48) confirmed the taxonomic affinity of *Bacteroides* species cultivars.

### Genomic and shotgun metagenomic sequencing.

A Covaris S220 ultrasonicator sheared 100 to 1,000 ng of DNA for each cultivar to 600 bp. Library construction followed protocols for the NuGEN Ovation Ultralow DNA library preparation or Illumina PCR-free library preparation prior to sequencing on a HiSeq Illumina platform. Metagenomic libraries were constructed for each of the 96 luminal (M) samples and three brush (GG) samples from the mucosa. Following DNA extraction, a NEBNext microbiome DNA enrichment kit reduced human genomic DNA contribution from the brush samples. DNA (final concentration of 0.01 to 0.2 ng/µl in 130 µl of 1× Tris-EDTA [TE] buffer) was sheared to base pairs. DNA shearing, end repair, adaptor ligation, and library amplification followed the NuGEN protocol. Pippin prepPrep (Sage Biosciences) selected for 170-bp inserts. Barcoded metagenomic libraries were sequenced on an Illumina platform to generate 97- to 113-bp paired-end reads. Read coverage ranged from ~7 million to ~146 million with an average of ~50 million reads/sample (see Table S3 in the supplemental material).

A BLAST filtering pipeline (https://github.com/meren/BLAST-filtering-pipeline) removed reads that matched the human reference genome at minimum length and identity of 50% and 90%, respectively (http://www.ncbi.nlm.nih.gov/assembly/2758/). MG-RAST (19) queried each read against the COG (clusters of orthologous groups) database (49) and M5nr database (50) and assigned a function/taxonomy if the query met the following criteria: maximum E value of 1e-5, minimum identity of 60%, and minimum alignment length of 15 amino acids.

### Shotgun metagenome assembly.

Illumina-utils (https://github.com/meren/illumina-utils) merged partially overlapping paired-end reads using “iu-merge-pairs” and retained joined sequences that displayed no mismatches within the overlapping regions. The flag “--enforce-Q30-check” eliminated paired-end reads if 66% of the bases in the first half of each had an average Q-score of less than Q30 (51). After pooling the data from samples taken throughout the longitudinal study of each patient, CLC Workbench v. 7.0.4 *de novo* assembled short reads into contigs with a word size, bubble size, and minimum contig length of 25, 165, and 2,000, respectively. Bowtie 2 (52) mapped the reads from each patient sample to the assembly generated for that patient. RAST (21) provided functional annotation and taxonomy assignments of contigs in the assemblies.

Anvi’o (20) generated interactive trees that facilitate the assignment of contigs to shotgun MAGs. The tetranucleotide frequency, congruence of coverage at each time point, taxonomy, GC content, and percentage of the contig covered defined the initial placement of each contig into a MAG. Anvi’o scanned all contigs for single-copy genes using HMMer v3.1b2 (53) and four single-copy gene collections (54–57) to estimate completion and contamination for each MAG. We scanned all contigs in each MAG for capsular polysaccharide (CPS) biosynthesis clusters (58) based upon similarity to gene models for *neuB* (http://pfam.xfam.org/family/NeuB), *wcbM*, (http://pfam.xfam.org/family/PF00483), capsular polysynthetase (http://pfam.xfam.org/family/PF05704), and *upxZ* (http://pfam.xfam.org/family/PF06603). Contigs were also scanned for *Bacteroides fragilis* toxin (BFT) using an HMM model for the N-terminal domain of fragilysin (http://pfam.xfam.org/family/PF16376).

### Cultivar genome assembly.

After quality filtering, the CLC Workbench v. 7.0.4 *de novo* assembled short reads into contigs. Mapping shotgun metagenomic reads from a patient sample to each *B. fragilis* cultivar genome assembly described the relative abundance of that cultivar in each patient visit sample. Reciprocal mapping of reads across cultivar genomes identified differences in gene content between the *Bacteroides* isolates, including the presence/absence and occurrence of multicopy genes. Read mapping shotgun metagenomic data sets from stool samples for 154 healthy humans against cultivar genomes from patients p-207, p-214, and n-216 identified genomic regions shared among *Bacteroides* from healthy patients in the Human Microbiome Project Data Analysis and Coordination Center (HMPDACC) (59, 60) and our cultivars. Bowtie 2 v2.0.5 (52) mapped short reads to contigs for visualizing coverage using anvi’o (52). The number of reads that mapped to the cultivar contigs divided by the total number of reads in the data set described the relative abundance of *B. fragilis* cultivars in each shotgun metagenomic data set. MAUVE aligned MAG and cultivar DNA sequences to determine the shared percent nucleotide identity.

### Statistical analysis.

ANOVA (analysis of variance) was used to test for significant differences in mean *Bacteroides* abundance among samples from N, W, I, and A visits. Estimates of *Bacteroides* abundance from GAST taxonomy, MG-RAST short-read annotation, and read recruitment to draft genomes served as input for the ANOVA. A posthoc Tukey’s HSD (honestly significant difference) test identified groups with significantly different means (61), and a Bartlett test determined whether the abundance variance differed significantly among groups (62). ANOVA tested for differences in the mean percent relative abundance of cultivar genomes from patient p-207, p-214, and n-216 in the 154 HMPDACC data sets and in samples from N, W, I, and A visits.

A linear mixed-effects model identified microbial groups that showed differences in abundance during inflammation. The model contained fixed effects for inflammation and days elapsed after the return of fecal flow into the pouch. Random effects of the model included patients and patient groups (n_patients versus p_patients). Coefficients and 97.5% confidence intervals for the model were estimated using the lmer function with restricted maximum likelihood (REML) in the R package “lme4” (63). The *P* values for fixed-effect model coefficients were adjusted using the Benjamin-Hochberg procedure to control the false-discovery rate (FDR). The relative abundance of each genus and oligotype served as input for the model.

Percent relative abundance matrices for GAST taxonomy, oligotypes, MG-RAST functional annotation, and RAST functional annotation of *Bacteroides* MAGs recovered from metagenomic samples provided input for bar plots, heat maps, and hierarchical cluster analysis. Hierarchical clustering for comparing microbiome composition employed the Ward (64) method on a Bray-Curtis (65) dissimilarity matrix. ADONIS, a nonparametric multivariate analysis of variance method tests for significant differences in beta diversity between specified groups and a null distribution that we created through permutation of the beta diversity matrix. ADONIS, a nonparametric multivariate analysis of variance method in the VEGAN package (66), tested for significant clustering of patient, pouch outcome (p_patients versus n_patients), and pouch condition (W, I, N, and A visits) variables for the GAST, MED, and MG-RAST input matrices generated with *betadiver*. ADONIS tested for significant clustering of the *Bacteroides* draft genome functional matrix for three variables: RAST species assignment, patient identifier (ID), and pouch outcome (p_patients versus n_patients). The *betadisper* function tested for differences in the homogeneity of group variances (67). R was used for all statistical analysis and visualization (68).

### Data availability.

The metagenomic and cultivar sequences are available through the NCBI Sequence Read Archive dbGaP (accession phs000262) and VAMPS hosts 16S sequences (https://vamps.mbl.edu) under project names HMP_200, HMP_202, HMP_204, HMP_207, HMP_208, HMP_209, HMP_210, HMP_211, HMP_ 212, HMP_213, HMP_214, HMP_215, HMP_216, HMP_217, HMP_218, HMP_219, HMP_423, HMP_427, HMP_500, HMP_502, and HMP_503.

## SUPPLEMENTAL MATERIAL

Figure S1  Relative abundance of taxa (GAST assignments). The heat map displays the percent relative abundance of genera within each sample based on 16S marker genes. GAST assigned taxonomy to each of the reads. The tree was constructed by average linkage using the Bray-Curtis dissimilarity matrix of the percent relative abundance matrix of genera within each sample. The names of the samples appear as leaves and are colored according to the condition of the pouch at the time the sample was collected. The name of the sample indicates the following: whether the patient developed pouchitis (p) or remained in a noninflamed condition (n) during the study, the identifier (ID) of the patient (e.g., 207), the visit number, if the sample was derived from the mucosa (GG) or lumen (M), and the number of days since pouch functionalization. Supplemental figures are available at doi:10.6084/m9.figshare.3851481. Download Figure S1, PDF file, 0.1 MB

Figure S2  Box-and-whisker plots for the percent relative abundance of *Bacteroides*. Samples were taken from pouches that never develop inflammation (N), were in an inflamed state (I), after antibiotic treatment (A), and without inflammation (W samples). The relative abundance was calculated using three distinct measures: 16S marker gene (GAST), short-read taxonomy matches to the M5nr database calculated by MG-RAST, and relative abundance of MAGs where the RAST closest neighbor matched *Bacteroides*. Supplemental figures are available at doi:10.6084/m9.figshare.3851481. Download Figure S2, PDF file, 0.1 MB

Figure S3  A cluster dendrogram based on MG-RAST functional annotation of each shotgun metagenomic data set. Clustering is based on Ward’s minimum variance of a Bray-Curtis dissimilarity matrix derived from the relative abundance of functions. A function was assigned if the read matched at 80% of the length and the hit achieved a maximum E value of 10^−15^. The leaves of the tree are labeled with the patient name (e.g., p-208), visit number, and days after the initialization of the pouch. The color of the sample name indicates the condition of the pouch as indicated by the key in the top left-hand corner of the figure. The relative abundance of each genus occurring at a minimum of 10% is displayed as a stacked bar plot below each sample. Supplemental figures are available at doi:10.6084/m9.figshare.3851481. Download Figure S3, PDF file, 0.1 MB

Figure S4  Functional cluster dendrogram of all *Bacteroides* MAGs. Functional classification of each genome is according to RAST, and the distance among the genomes was calculated using Ward’s minimum variance of a Bray-Curtis dissimilarity matrix derived from the relative abundance of functions. The number of HMM hits for each of the genes within the CPS and regulatory elements for each genome is summarized in the corresponding table. The color of the genome name indicates whether the sample was isolated from a pouchitis (red) or nonpouchitis (blue) patient. The background color in each cell of the table reflects the amount in the cell relative to other cells in the same column. White is low and orange is high. Supplemental figures are available at doi:10.6084/m9.figshare.3851481. Download Figure S4, PDF file, 0.1 MB

Figure S5  Cultivar detection throughout longitudinal sampling using shotgun metagenomic sequences. To determine whether a cultivar isolated from an individual was present throughout the longitudinal sampling, we mapped each of the shotgun metagenomic data sets for the patient back to the cultivar assembly. The figure displays mean coverage for 2-kbp sections of the ~5-Mbp assembly as separate bar plots for each of the longitudinal samples in chronological order with the age (days) of the active pouch shown on the right-hand side of the figure. All samples taken come from luminal pouch samples unless indicated as “mucosal” (using a brush sampling protocol). The color of the bar indicates the condition of the patient at the time of sample collection: without inflammation (orange), inflamed pouch (red), after (post) antibiotic treatment (purple), or a nonpouchitis patient sample (light green). The results of mapping shotgun sequences of the cultivar back to the assembly are shown as black histograms, and the percent GC content of the 2-kbp sections are shown in dark green. A red bar at the bottom of the figure shows the location of the transcriptional regulatory element *upxY* for capsular polysaccharide biosynthesis based upon HMM models. Supplemental figures are available at doi:10.6084/m9.figshare.3851481. Download Figure S5, PDF file, 2.29 MB

Table S1  Clinical metadata for each sample. The sample name indicates pouchitis (p) or nonpouchitis (n) patients, patient ID, visit number, luminal aspirate (M) versus mucosal-brush (GG) sampling site, and the number of days after pouch functionalization. The condition of the pouch and the results of clinical examination of the patient and what treatment (if any) was prescribed are shown. Supplemental tables are available at doi:10.6084/m9.figshare.3851478 Table S1, PDF file, 0.04 MB

Table S2  Summary of linear mixed-effects model results. The coefficients predicted by the linear mixed-effects model for relative abundance of each genus and oligotype in this study are shown. The 97.5% confidence intervals for each coefficient are reported as coef_lwr (coef stands for coefficient, and lwr stands for lower) (2.5%) and coef_upr (upr stands for upper) (97.5%). The uncorrected *P* value and FDR-corrected *P* value (adj_pvalue [for adjusted *P* value]) are reported for each coefficient. Supplemental tables are available at doi:10.6084/m9.figshare.3851478 Table S2, PDF file, 0.1 MB

Table S3  Abundant *Bacteroides* oligotypes and MAGs isolated from pouchitis patients during inflammation. The letters a to e indicate unique *Bacteroides* MED oligotypes detected during inflammation with “na” indicating samples not available from visits during inflammation. Taxonomy of the oligotype is based on similarity to hits in the NCBI RefSeq rRNA database. The table reports the most abundant *Bacteroides* MAG and the percent recruitment during inflammation and MAG taxonomy assignments based on similarity to genomes contained in the RAST database. Supplemental tables are available at doi:10.6084/m9.figshare.3851478 Table S3, PDF file, 0.03 MB

Table S4  Summary of MAGs recovered from each patient. The summary includes the patient ID, unique MAG ID, taxonomy, summary of the MAG size, common assembly statistics, and completion/contamination based on three bacterial single-copy core gene references. Taxonomy is based on the most common hit of the MAG contigs to genomes in the RAST database. Supplemental tables are available at doi:10.6084/m9.figshare.3851478. The supplemental material at doi:10.6084/m9.figshare.3851364 includes anvi'o profiles to visualize and re-analyze metagenomes for each patient, and MAGs reported in the manuscript. Table S4, PDF file, 0.1 MB

Table S5  Functions of high- and low-coverage regions of p214 *B. fragilis* genome within HMPDACC samples. The table is supplemental to Fig. S6 and details the functions identified within the rainbow-highlighted regions of the figure. The number recorded in the first column “bin” corresponds with the number adjacent to the highlighted region of the figure. The rows adjacent to each bin include the RAST assigned function, the identity of the contig containing the function, start and stop position within the contig, and orientation of the sequence (f for forward, r for reverse). Supplemental tables are available at doi:10.6084/m9.figshare.3851478. Supplemental figures are available at doi:10.6084/m9.figshare.3851481 Table S5, PDF file, 0.05 MB
