# Supplementary material for: Patient-Specific Bacteroides Genome Variants in Pouchitis
Source: mBio. 2016 Nov 15;7(6):e01713-16. doi: 10.1128/mBio.01713-16 (PMC5111406; doi:10.1128/mBio.01713-16)
Supplement: Figure S1 — Relative abundance of taxa (GAST assignments). The heat map displays the percent relative abundance of genera within each sample based on 16S marker genes. GAST assigned taxonomy to each of the reads. The tree was constructed by average linkage using the Bray-Curtis dissimilarity matrix of the percent relative abundance matrix of genera within each sample. The names of the samples appear as leaves and are colored according to the condition of the pouch at the time the sample was collected. The name of the sample indicates the following: whether the patient developed pouchitis (p) or remained in a noninflamed condition (n) during the study, the identifier (ID) of the patient (e.g., 207), the visit number, if the sample was derived from the mucosa (GG) or lumen (M), and the number of days since pouch functionalization. Supplemental figures are available at doi:10.6084/m9.figshare.3851481. Download [file mbo005163055sf1.pdf]

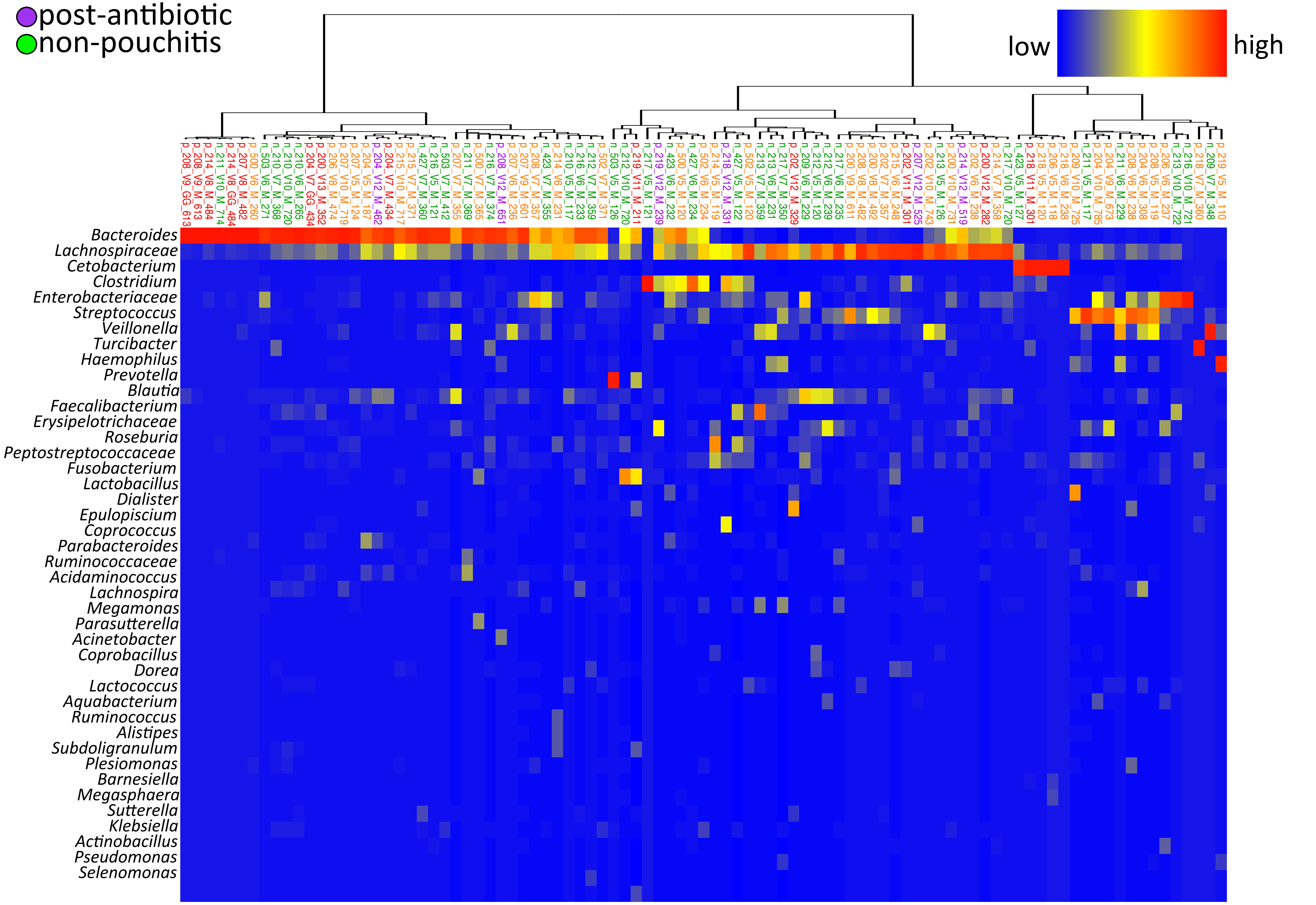
inflamed
without inflammation
post-antibiotic
non-pouchitis
Percent Relative Abundance
low
high
Bacteroides
Lachnospiraceae
Cetobacterium
Clostridium
Enterobacteriaceae
Streptococcus
Veillonella
Turcibacter
Haemophilus
Prevotella
Blautia
Faecalibacterium
Erysipelotrichaceae
Roseburia
Peptostreptococcaceae
Fusobacterium
Lactobacillus
Dialister
Epulopiscium
Coprococcus
Parabacteroides
Ruminococcaceae
Acidaminococcus
Lachnospira
Megamonas
Parasutterella
Acinetobacter
Coprobacillus
Dorea
Lactococcus
Aquabacterium
Ruminococcus
Alistipes
Subdoligranulum
Plesiomonas
Barnesiella
Megasphaera
Sutterella
Klebsiella
Actinobacillus
Pseudomonas
Selenomonas

Figure S1.
