# Supplementary material for: Patient-Specific Bacteroides Genome Variants in Pouchitis
Source: mBio. 2016 Nov 15;7(6):e01713-16. doi: 10.1128/mBio.01713-16 (PMC5111406; doi:10.1128/mBio.01713-16)
Supplement: Figure S2 — Box-and-whisker plots for the percent relative abundance of Bacteroides. Samples were taken from pouches that never develop inflammation (N), were in an inflamed state (I), after antibiotic treatment (A), and without inflammation (W samples). The relative abundance was calculated using three distinct measures: 16S marker gene (GAST), short-read taxonomy matches to the M5nr database calculated by MG-RAST, and relative abundance of MAGs where the RAST closest neighbor matched Bacteroides. Supplemental figures are available at doi:10.6084/m9.figshare.3851481. Download [file mbo005163055sf2.pdf]

Figure S2.

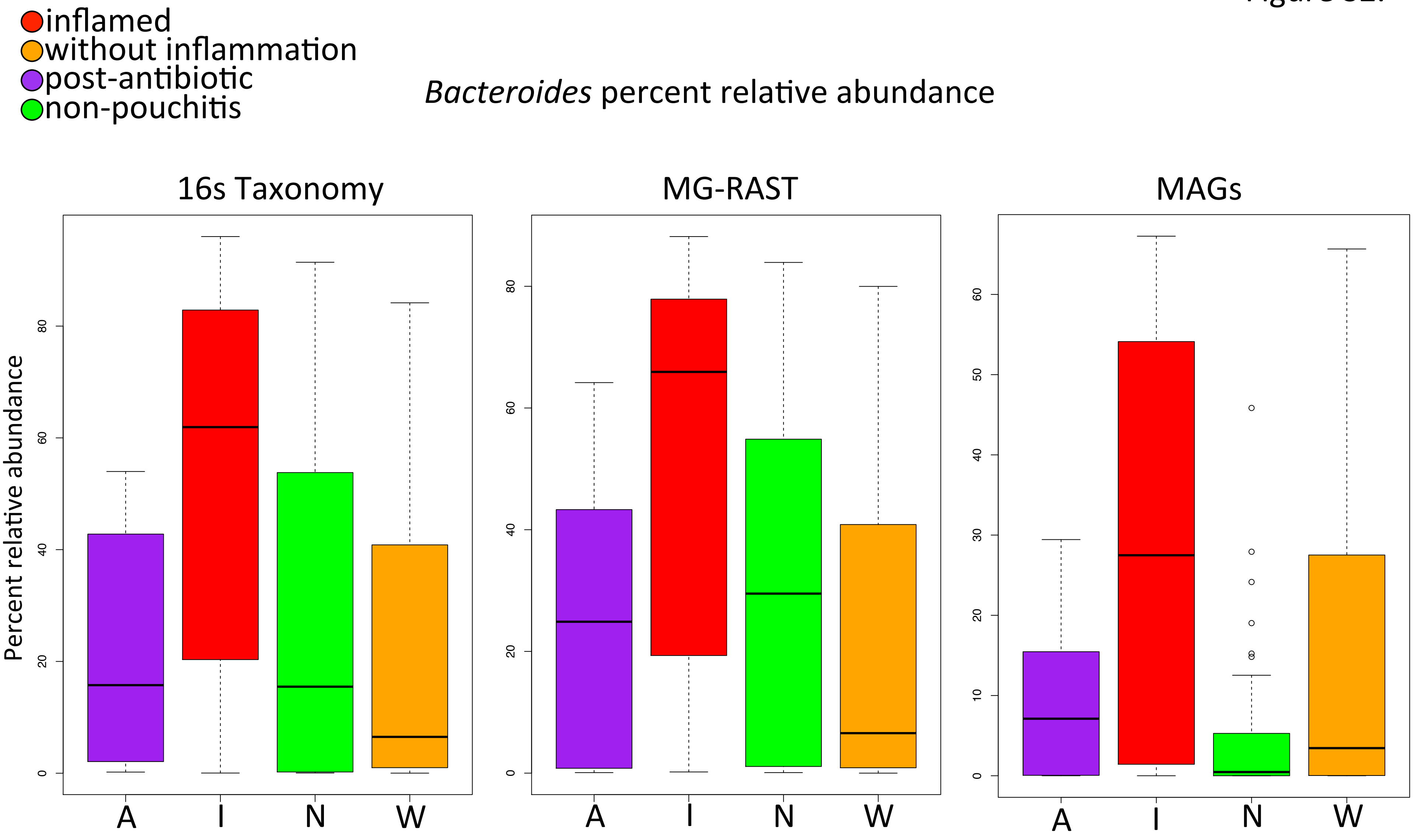

inflamed
without inflammation
post-antibiotic
non-pouchitis
Bacteroides percent relative abundance
16s Taxonomy
MG-RAST
MAGs
Percent relative abundance
0
20
40
60
80
10
30
50
A
I
N
W
