# Supplementary figures and images for: Patient-Specific Bacteroides Genome Variants in Pouchitis
Source: mBio. 2016 Nov 15;7(6):e01713-16. doi: 10.1128/mBio.01713-16 (PMC5111406; doi:10.1128/mBio.01713-16)

Figure S3.

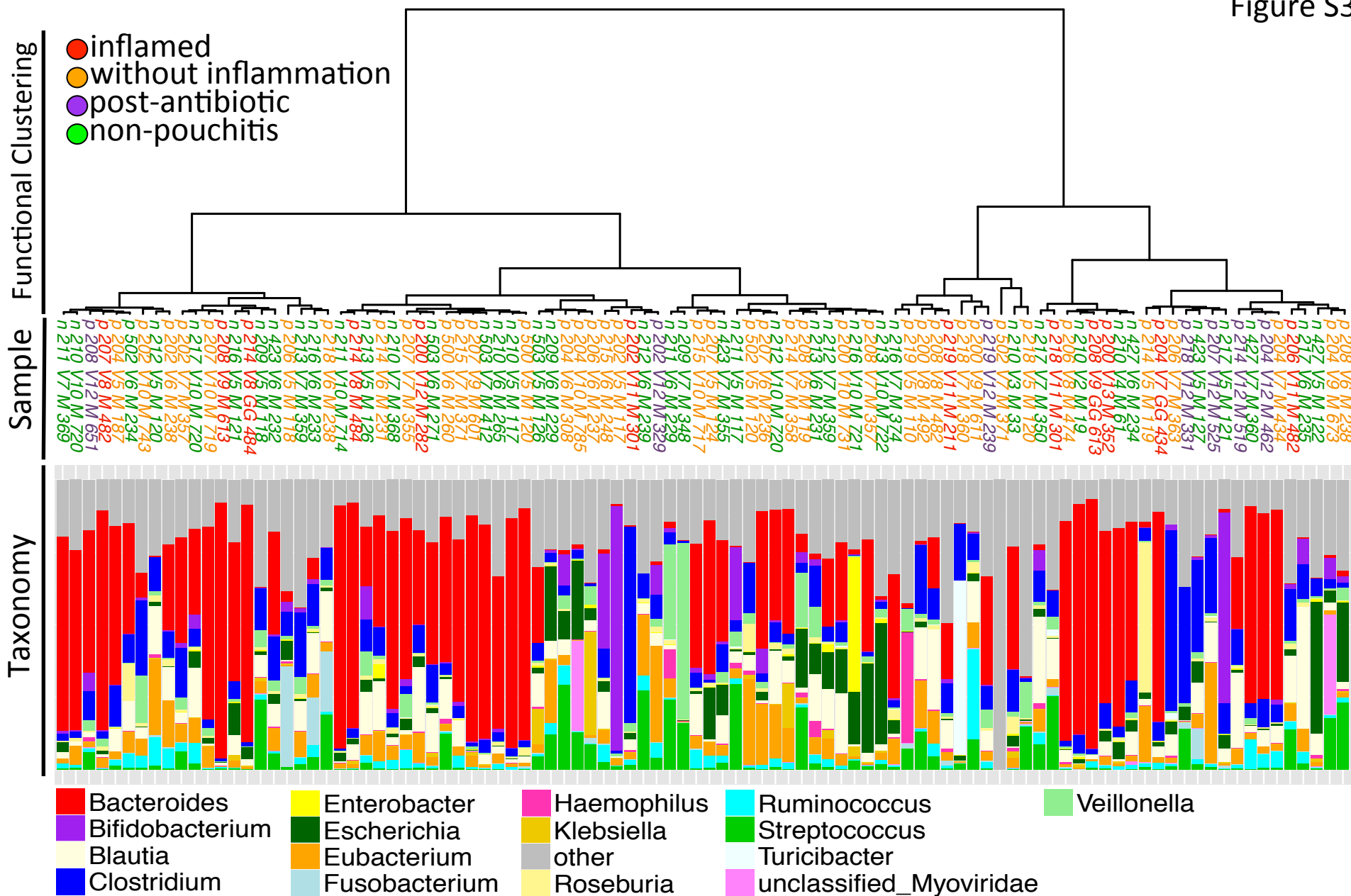

Supplement: Figure S3 — A cluster dendrogram based on MG-RAST functional annotation of each shotgun metagenomic data set. Clustering is based on Ward’s minimum variance of a Bray-Curtis dissimilarity matrix derived from the relative abundance of functions. A function was assigned if the read matched at 80% of the length and the hit achieved a maximum E value of 10−15. The leaves of the tree are labeled with the patient name (e.g., p-208), visit number, and days after the initialization of the pouch. The color of the sample name indicates the condition of the pouch as indicated by the key in the top left-hand corner of the figure. The relative abundance of each genus occurring at a minimum of 10% is displayed as a stacked bar plot below each sample. Supplemental figures are available at doi:10.6084/m9.figshare.3851481. Download [file mbo005163055sf3.pdf]

Figure S4.

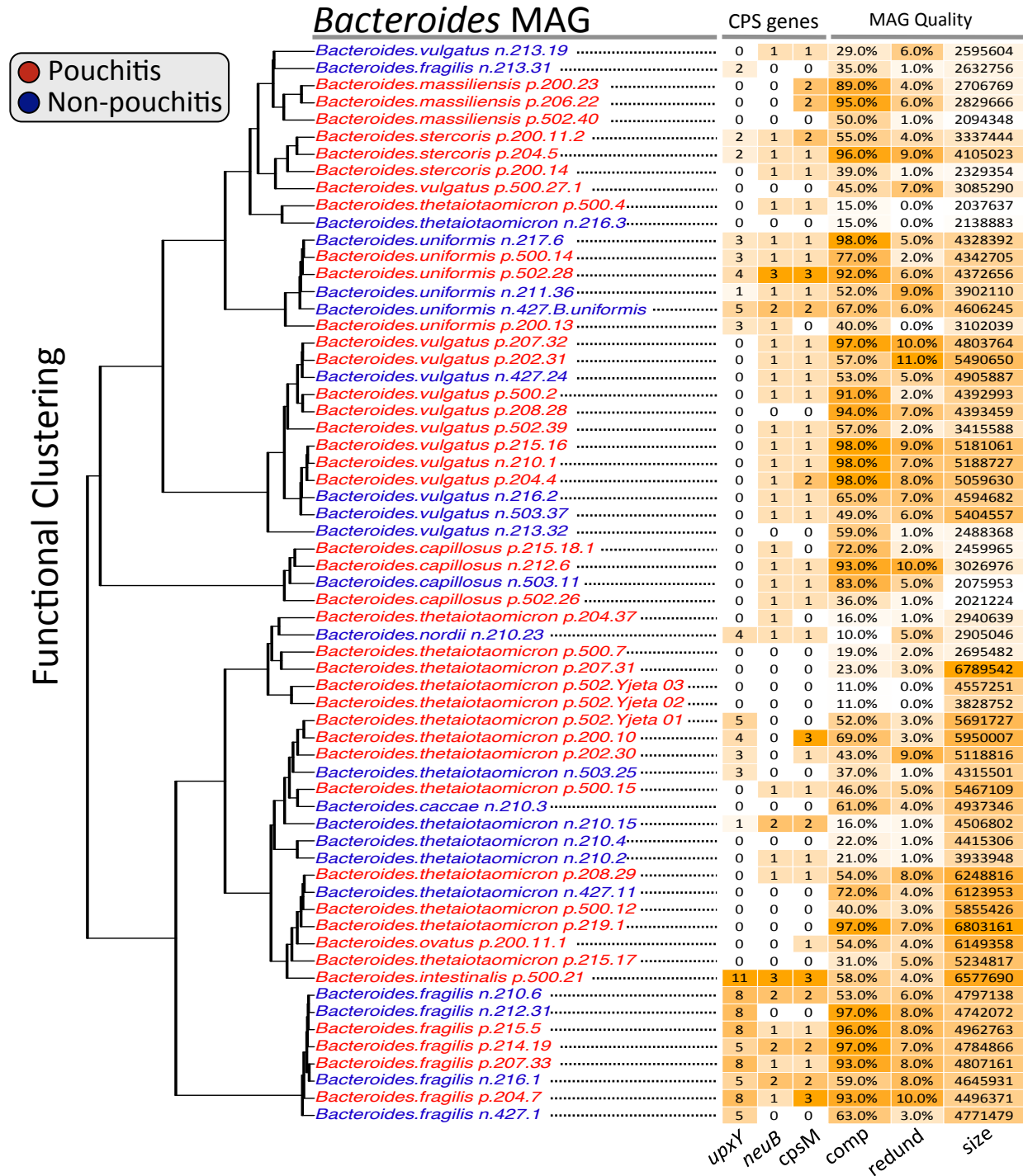

Supplement: Figure S4 — Functional cluster dendrogram of all Bacteroides MAGs. Functional classification of each genome is according to RAST, and the distance among the genomes was calculated using Ward’s minimum variance of a Bray-Curtis dissimilarity matrix derived from the relative abundance of functions. The number of HMM hits for each of the genes within the CPS and regulatory elements for each genome is summarized in the corresponding table. The color of the genome name indicates whether the sample was isolated from a pouchitis (red) or nonpouchitis (blue) patient. The background color in each cell of the table reflects the amount in the cell relative to other cells in the same column. White is low and orange is high. Supplemental figures are available at doi:10.6084/m9.figshare.3851481. Download [file mbo005163055sf4.pdf]
