# Supplementary material for: Patient-Specific Bacteroides Genome Variants in Pouchitis
Source: mBio. 2016 Nov 15;7(6):e01713-16. doi: 10.1128/mBio.01713-16 (PMC5111406; doi:10.1128/mBio.01713-16)

Bacteroides fragilis : p214- cultivar : isolated from day 484 brush sample : genome size = 5.14Mb

Fig S5.

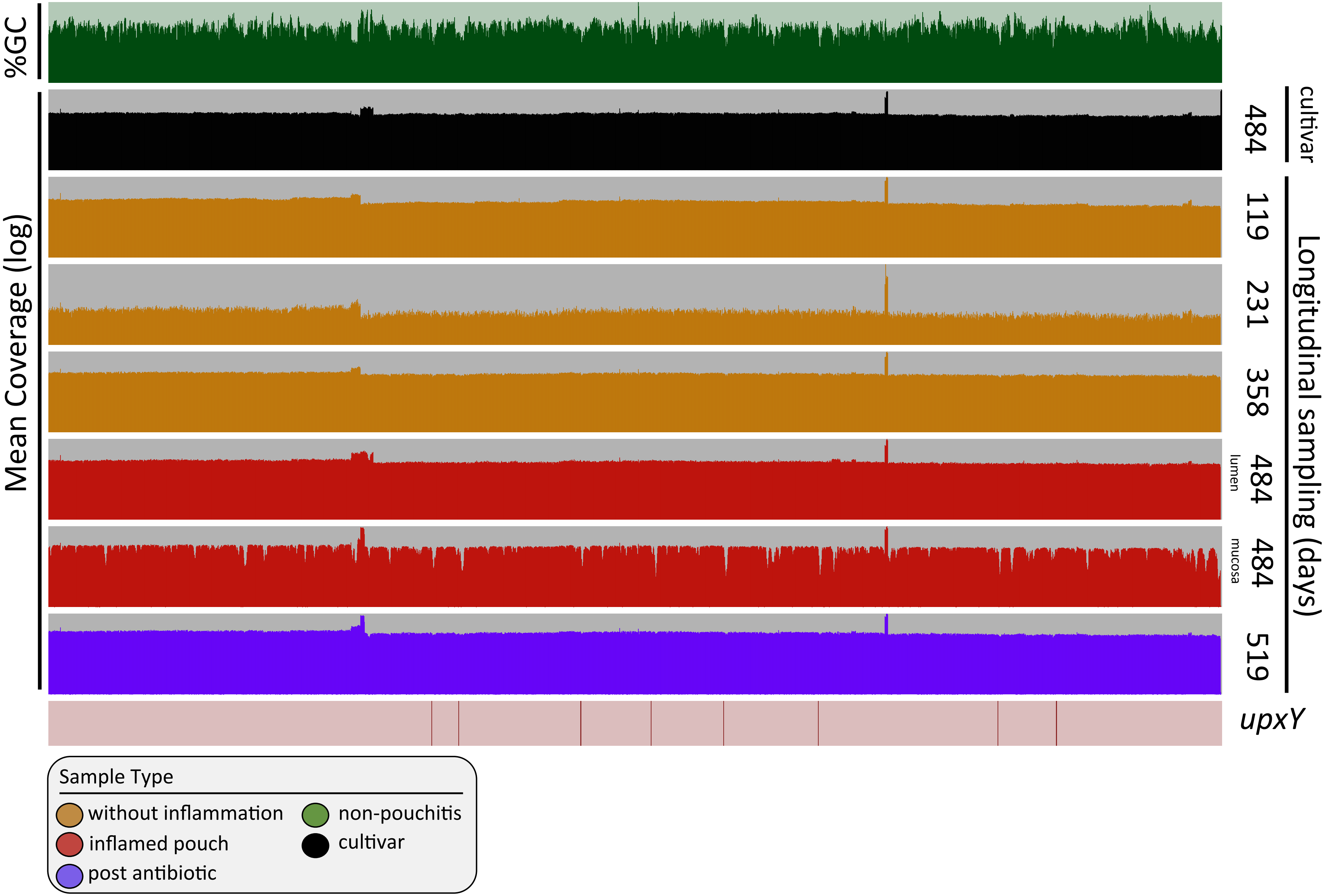

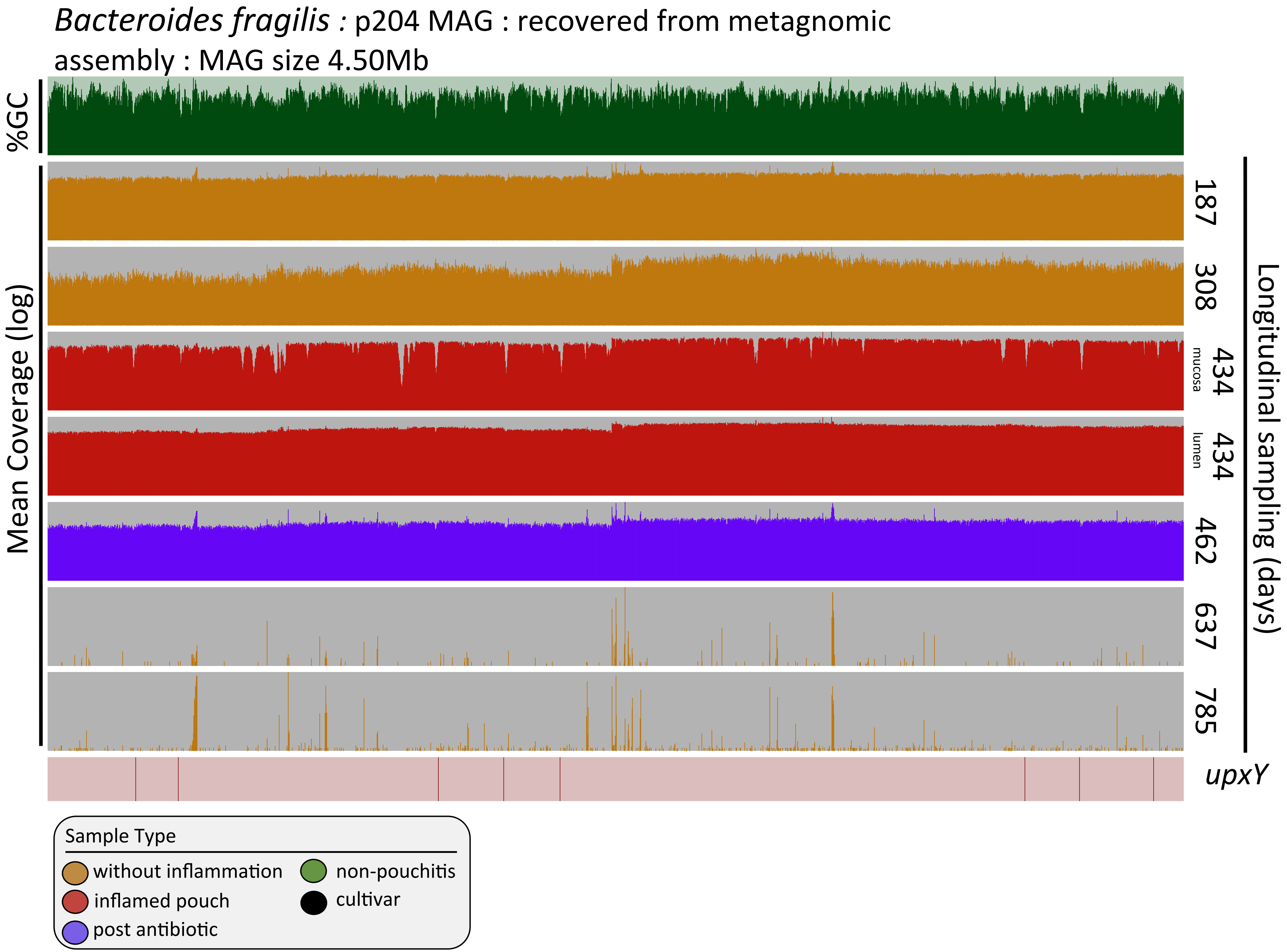

Bacteroides fragilis : p204 MAG : recovered from metagnomic assembly : MAG size 4.50Mb
%GC
Mean Coverage (log)
187
308
mucosa
434
lumen
434
462
637
785
Longitudinal sampling (days)
upxY
Sample Type
without inflammation
non-pouchitis
inflamed pouch
cultivar
post antibiotic

Bacteroides fragilis : p207 - cultivar : isolated from day 482 brush sample : genome size = 5.00Mb

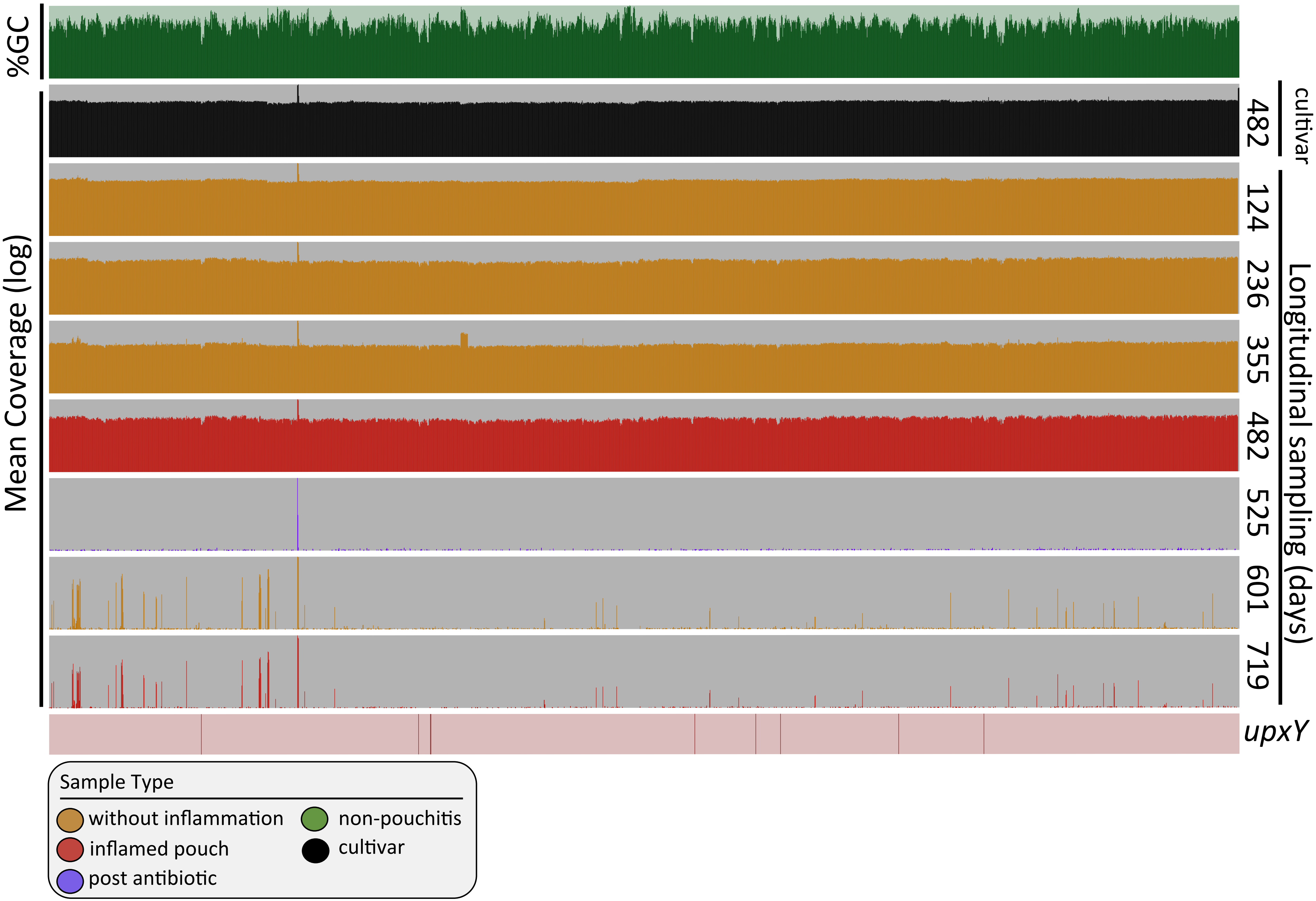

Bacteroides thetaiotaomicron : p219 - cultivar : isolated from day 239

brush sample : genome size = 6.99Mb

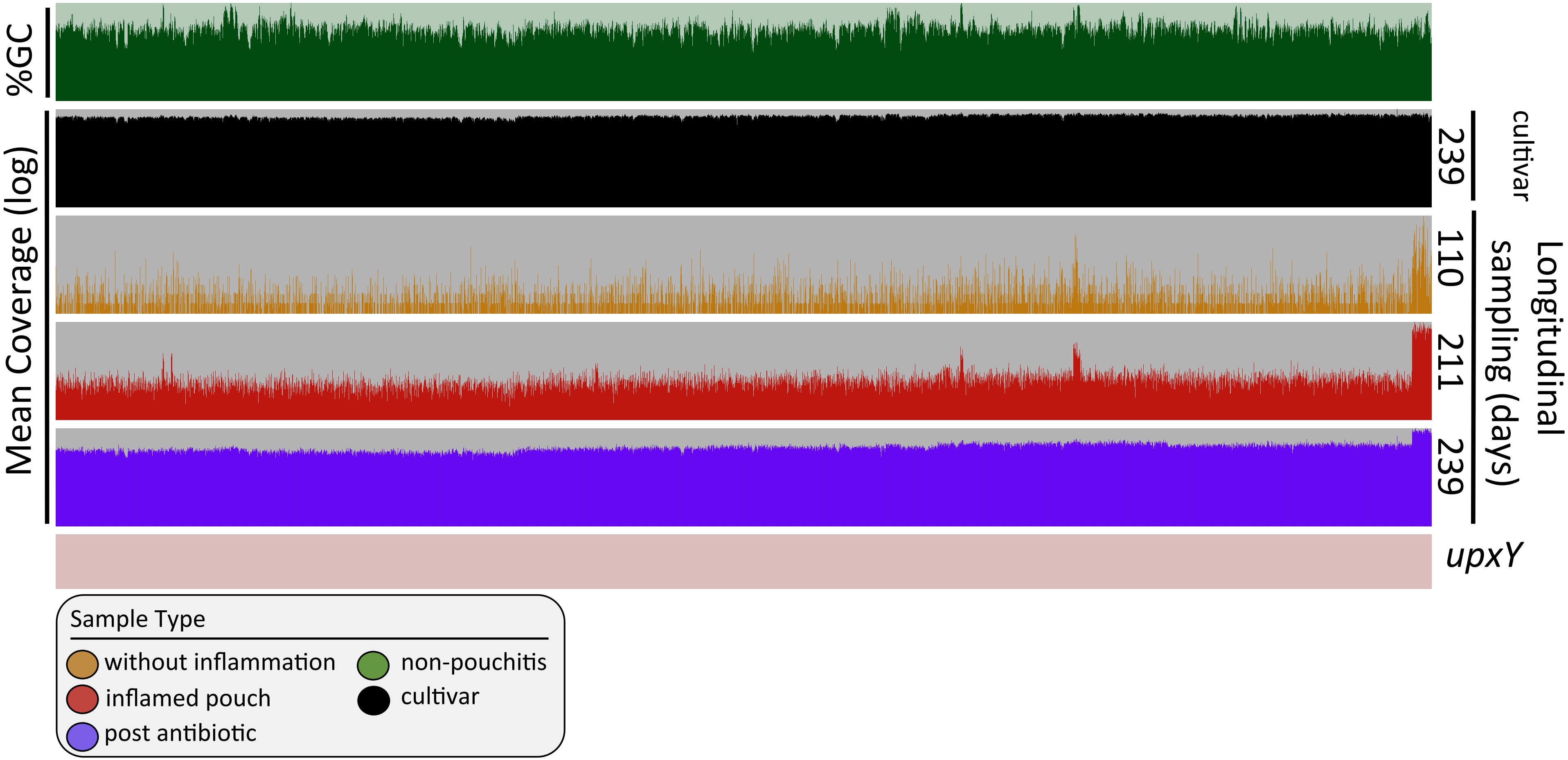

Bacteroides fragilis : p215 - cultivar : isolated from day 717 brush sample : genome size = 5.00Mb

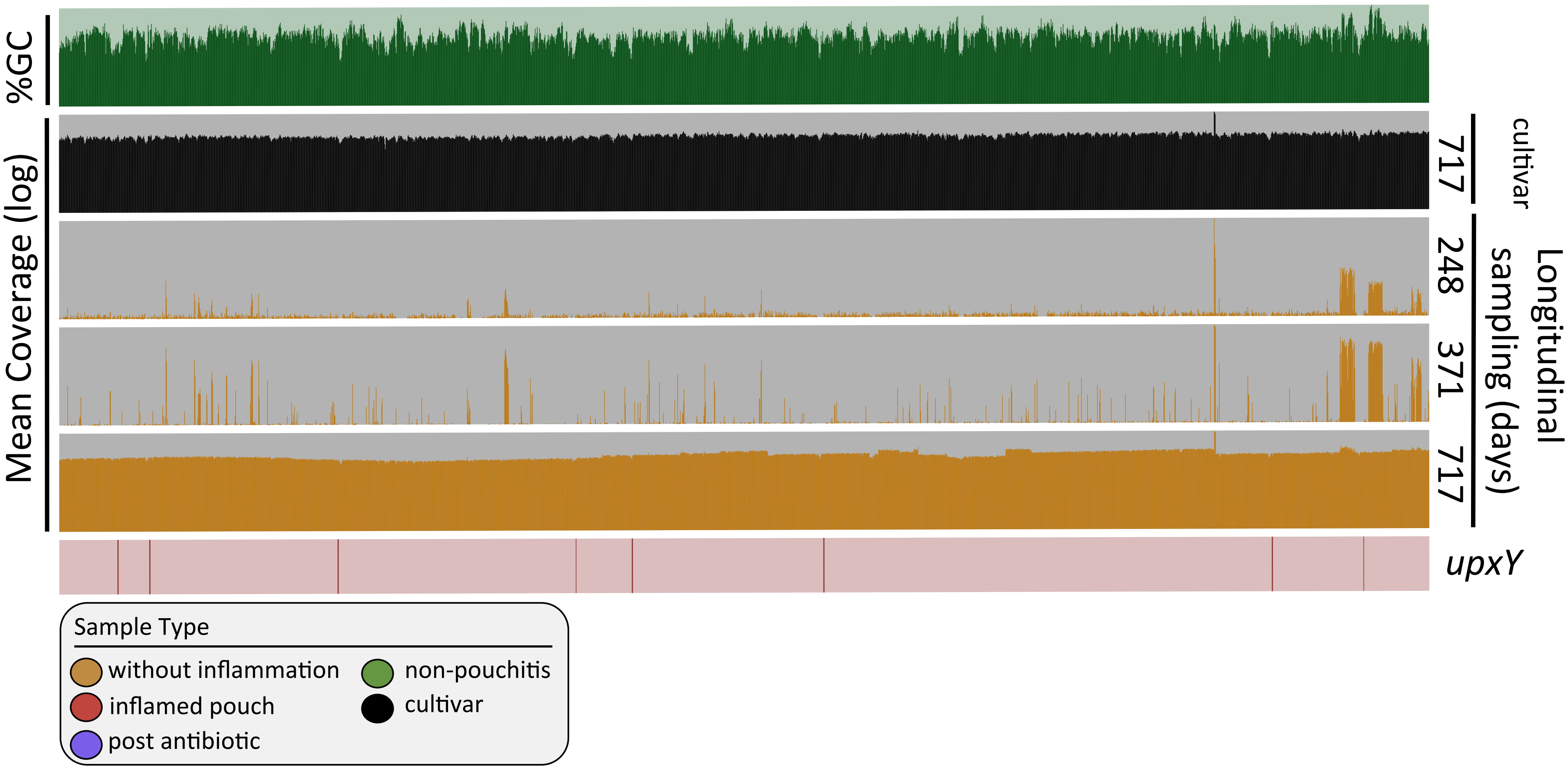

Bacteroides fragilis : p216 - cultivar : isolated from day 121 brush sample : genome size = 5.00Mb

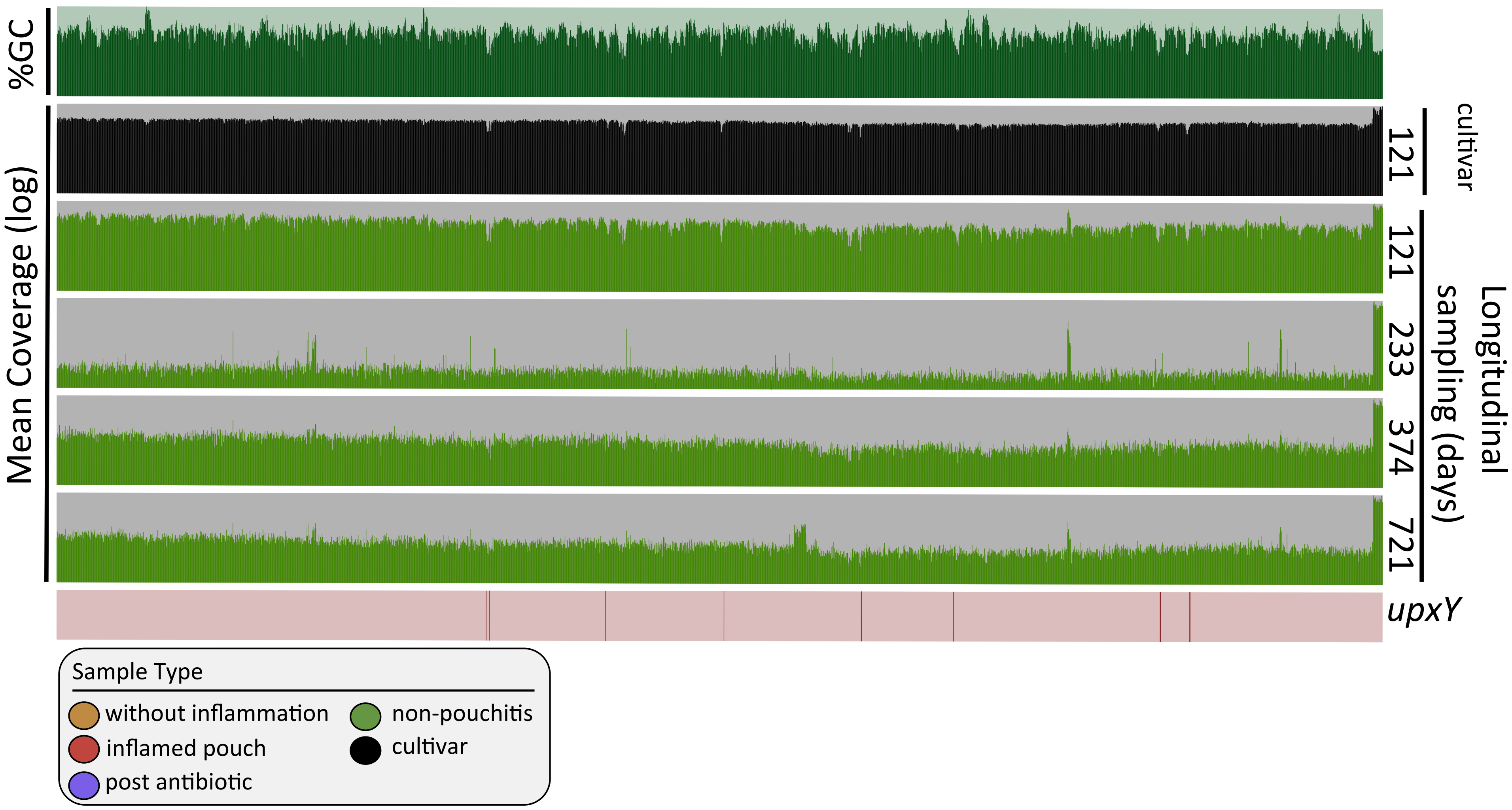

Supplement: Figure S5 — Cultivar detection throughout longitudinal sampling using shotgun metagenomic sequences. To determine whether a cultivar isolated from an individual was present throughout the longitudinal sampling, we mapped each of the shotgun metagenomic data sets for the patient back to the cultivar assembly. The figure displays mean coverage for 2-kbp sections of the ~5-Mbp assembly as separate bar plots for each of the longitudinal samples in chronological order with the age (days) of the active pouch shown on the right-hand side of the figure. All samples taken come from luminal pouch samples unless indicated as “mucosal” (using a brush sampling protocol). The color of the bar indicates the condition of the patient at the time of sample collection: without inflammation (orange), inflamed pouch (red), after (post) antibiotic treatment (purple), or a nonpouchitis patient sample (light green). The results of mapping shotgun sequences of the cultivar back to the assembly are shown as black histograms, and the percent GC content of the 2-kbp sections are shown in dark green. A red bar at the bottom of the figure shows the location of the transcriptional regulatory element upxY for capsular polysaccharide biosynthesis based upon HMM models. Supplemental figures are available at doi:10.6084/m9.figshare.3851481. Download [file mbo005163055sf5.pdf]
