# Supplementary material for: Patient-Specific Bacteroides Genome Variants in Pouchitis
Source: mBio. 2016 Nov 15;7(6):e01713-16. doi: 10.1128/mBio.01713-16 (PMC5111406; doi:10.1128/mBio.01713-16)
Supplement: Table S1 — Clinical metadata for each sample. The sample name indicates pouchitis (p) or nonpouchitis (n) patients, patient ID, visit number, luminal aspirate (M) versus mucosal-brush (GG) sampling site, and the number of days after pouch functionalization. The condition of the pouch and the results of clinical examination of the patient and what treatment (if any) was prescribed are shown. Supplemental tables are available at doi:10.6084/m9.figshare.3851478 [file mbo005163055st1.pdf]

Table S1. Clinical metadata for each sample

Sample Name	Age	Gender	Race	Smoker	Condition	Clinician Diagnosis	Clinical sx score	Endoscopic score	Histologic score	Total PDAI	Treatment
p_200_V12_M_282	23	M	Hispanic	No	inflamation		0	5	2	7	
p_200_V13_M_352	23	M	Hispanic	No	inflamation	C diff	4	6	3	13	Flagyl for C diff infection
p_200_V8_M_492	23	M	Hispanic	No	without inflamation		0	2	4	6	VSL#3 given
p_200_V9_M_611	23	M	Hispanic	No	without inflamation		0	2	?	2	Stopped VSL#3
p_200_V10_M_731	23	M	Hispanic	No	without inflamation		1	3	2	6	
p_202_V5_M_117	54	M	Caucasian	ex	without inflamation	Granulomas in pouch	0	0	3	3	
p_202_V6_M_238	54	M	Caucasian	ex	without inflamation		0	0	4	4	
p_202_V11_M_301	54	M	Caucasian	ex	inflamation	Pouchitis	3	5	2	10	Cipro
p_202_V12_M_329	54	M	Caucasian	ex	antibiotic		0	0	0	0	VSL#3
p_202_V7_M_357	54	M	Caucasian	ex	without inflamation		0	1	2	3	VSL#3
p_202_V10_M_743	54	M	Caucasian	ex	without inflamation		2	0	2	4	VSL#3
p_204_V5_M_187	25	M	Caucasian	No	without inflamation		0	2	2	4	
p_204_V6_M_308	25	M	Caucasian	No	without inflamation		0	1	0	1	
p_204_V7_M_434	25	M	Caucasian	No	inflamation	Pouchitis	2	6	1	9	Cipro
p_204_V7_GG_434	25	M	Caucasian	No	inflamation	Pouchitis	2	6	1	9	Cipro
p_204_V12_M_462	25	M	Caucasian	No	antibiotic		0	5	0	5	
p_204_V9_M_673	25	M	Caucasian	No	without inflamation		0	3	0	3	
p_204_V10_M_785	25	M	Caucasian	No	without inflamation		0	1	0	1	
p_206_V5_M_118	19	M	Caucasian	No	without inflamation	mild pouch inflammation	0	3	1	4	
p_206_V6_M_237	19	M	Caucasian	No	without inflamation	few scattered ulcers	0	1	0	1	
p_206_V7_M_363	19	M	Caucasian	No	without inflamation		0	0	0	0	
p_206_V8_M_474	19	M	Caucasian	No	without inflamation	patchy mild erythema	0	1	0	1	
p_206_V11_M_482	19	M	Caucasian	No	inflamation	Pouchitis	4	5	0	9	Cipro
p_207_V5_M_124	31	M	Caucasian	ex	without inflamation		2	1	0	3	
p_207_V6_M_236	31	M	Caucasian	ex	without inflamation		0	1	1	2	
p_207_V7_M_355	31	M	Caucasian	ex	without inflamation		1	1	0	2	
p_207_V8_M_482	31	M	Caucasian	ex	inflamation	Pouchitis	2	4	2	8	Cipro
p_207_V12_M_525	31	M	Caucasian	ex	antibiotic		0	1	0	1	
p_207_V9_M_601	31	M	Caucasian	ex	without inflamation		1	1	0	2	
p_207_V10_M_719	31	M	Caucasian	ex	inflamation	Pouchitis	3	6	2	11	Cipro
p_208_V5_M_119	65	M	Caucasian	ex	without inflamation		0	0	1	1	
p_208_V6_M_238	65	M	Caucasian	ex	without inflamation		1	0	1	2	
p_208_V7_M_357	65	M	Caucasian	ex	without inflamation		0	0	0	0	
p_208_V8_M_482	65	M	Caucasian	ex	without inflamation		1	0	0	1	
p_208_V9_GG_613	65	M	Caucasian	ex	inflamation	Pouchitis	4	1	2	7	Cipro
p_208_V9_M_613	65	M	Caucasian	ex	inflamation	Pouchitis	4	1	2	7	
p_208_V12_M_651	65	M	Caucasian	ex	antibiotic		3	1	1	5	Had abx 2/1/12-2/15/12 for pouchitis
n_209_V6_M_229	50	M	Caucasian	No	non-pouchitis		0	1	1	2	Levaquin 1/14/11-1/19/11;Oral steroid for lungs
n_209_V7_M_348	50	M	Caucasian	No	non-pouchitis	small perianal fisutula	0	0	0	0	Cipro 4/26-5/15 Augmentin 4/19/11-4/26/11
n_209_V5_M_118	50	M	Caucasian	No	non-pouchitis		0	0	0	0	
n_210_V2_M_19	45	M	Caucasian	ex	non-pouchitis		0	0	1	1	prednisone 5mg daily
n_210_V3_M_33	45	M	Caucasian	ex	non-pouchitis		0	0	0	0	prednisone 5mg daily
n_210_V4_M_61	45	M	Caucasian	ex	non-pouchitis	inflammatory polyps near the suture line in distal pouch	1	1	0	2	prednisone 2.5mg daily
n_210_V5_M_117	45	M	Caucasian	ex	non-pouchitis	mild inflammation, some granularity, small aphthae	0	2	1	3	
n_210_V6_M_265	45	M	Caucasian	ex	non-pouchitis	few scattered aphthae in the pouch	0	1	0	1	Azithromycin 1/15/11-1/20/11
n_210_V7_M_368	45	M	Caucasian	ex	non-pouchitis		0	0	0	0	
n_210_V10_M_720	45	M	Caucasian	ex	non-pouchitis	mild granularity in the pouch	0	1	0	1	
n_211_V5_M_117	32	M	Caucasian	No	non-pouchitis		0	0	0	0	
n_211_V6_M_229	32	M	Caucasian	No	non-pouchitis		0	0	0	0	Prilosec
n_211_V7_M_369	32	M	Caucasian	No	non-pouchitis		0	0	2	2	Prilosec
n_211_V10_M_714	32	M	Caucasian	No	non-pouchitis		0	0	1	1	
n_212_V5_M_120	32	F	Caucasian	No	non-pouchitis		0	0	0	0	Canasa
n_212_V6_M_239	32	F	Caucasian	No	non-pouchitis		0	0	0	0	Canasa
n_212_V7_M_359	32	F	Caucasian	No	non-pouchitis		0	0	0	0	Canasa
n_212_V10_M_720	32	F	Caucasian	No	non-pouchitis		0	0	1	1	Canasa
n_213_V5_M_126	37	M	Caucasian	No	non-pouchitis		0	0	1	1	Immodium
n_213_V6_M_231	37	M	Caucasian	No	non-pouchitis		0	0	0	0	Immodium
n_213_V7_M_359	37	M	Caucasian	No	non-pouchitis		1	0	1	2	Immodium, VSL#3 as needed, GasX
n_213_V10_M_722	37	M	Caucasian	No	non-pouchitis		1	0	0	1	Immodium
p_214_V5_M_119	29	F	Caucasian	No	without inflamation		0	1	1	2	VSL#3
p_214_V6_M_231	29	F	Caucasian	No	without inflamation		0	0	0	0	VSL#3
p_214_V7_M_358	29	F	Caucasian	No	without inflamation		0	0	0	0	VSL#3
p_214_V8_GG_484	29	F	Caucasian	No	inflamation	diagnosed pouchitis	2	2	0	4	Cipro
p_214_V8_M_484	29	F	Caucasian	No	inflamation	diagnosed pouchitis	2	2	0	4	Cipro
p_214_V12_M_519	29	F	Caucasian	No	antibiotic	few scatterd ulcers	0	1	0	1	
p_215_V6_M_248	21	F	Af-Am	Yes	without inflamation	mild cuff inflammation	1	0	0	1	
p_215_V7_M_371	21	F	Af-Am	Yes	without inflamation		0	1	3	4	
p_215_V10_M_717	21	F	Af-Am	Yes	without inflamation		1	0	1	2	
n_216_V5_M_121	42	M	Caucasian	ex	non-pouchitis	rectal cuff lined with ulcers	3	0	0	3	Budesonide/Canasa suppository
n_216_V6_M_233	42	M	Caucasian	ex	non-pouchitis	rectal cuff edematous	1	0	0	1	steroid cream
n_216_V7_M_374	42	M	Caucasian	ex	non-pouchitis	mild cuffitis	2	0	0	2	
n_216_V10_M_721	42	M	Caucasian	ex	non-pouchitis	erythema at cuff	0	0	0	0	
n_217_V5_M_121	45	M	Caucasian	ex	non-pouchitis	few ulcers and erythema along suture line	0	0	0	0	
n_217_V6_M_235	45	M	Caucasian	ex	non-pouchitis	few ulcers along suture line	0	0	0	0	
n_217_V7_M_350	45	M	Caucasian	ex	non-pouchitis		0	1	0	1	
n_217_V10_M_720	45	M	Caucasian	ex	non-pouchitis		0	0	0	0	
p_218_V5_M_120	23	M	Caucasian	No	without inflamation	few scattered ulcers throughout the pouch	0	1	0	1	
p_218_V6_M_238	23	M	Caucasian	No	without inflamation	scattered ulcers	0	1	0	1	
p_218_V11_M_301	23	M	Caucasian	No	inflamation	pouchitis diagnosed	0	0	0	0	
p_218_V12_M_331	23	M	Caucasian	No	antibiotic	few aphthe in pouch	0	0	0	0	
p_218_V7_M_360	23	M	Caucasian	No	without inflamation	some scattered aphthae	0	1	0	1	
p_219_V5_M_110	23	M	Caucasian	No	without inflamation		0	0	0	0	
p_219_V11_M_211	23	M	Caucasian	No	inflamation	Pre-pouch ileum had scattered aphthous ulcers, path no	5	2	0	7	
p_219_V12_M_239	23	M	Caucasian	No	antibiotic	path consistent with pouchitis	0	1	0	1	
n_423_V5_M_127	53	M	Caucasian	no	non-pouchitis		0	1	0	1	
n_423_V6_M_232	53	M	Caucasian	no	non-pouchitis		0	0	0	0	
n_423_V7_M_355	53	M	Caucasian	no	non-pouchitis		0	0	1	1	
n_427_V5_M_122	30	F	Caucasian	No	non-pouchitis		0	0	1	1	
n_427_V6_M_234	30	F	Caucasian	No	non-pouchitis		0	0	2	2	
n_427_V7_M_360	30	F	Caucasian	No	non-pouchitis		0	1	2	3	
p_500_V5_M_120	28	M	Caucasian	No	without inflamation		1	0	2	3	
p_500_V6_M_260	28	M	Caucasian	No	without inflamation		0	0	2	2	
p_500_V7_M_365	28	M	Caucasian	No	without inflamation		0	1	0	0	
p_502_V5_M_120	39	F	Caucasian	ex	without inflamation		0	1	2	3	
p_502_V6_M_234	39	F	Caucasian	ex	without inflamation		0	0	1	1	
p_502_V7_M_371	39	F	Caucasian	ex	without inflamation		0	0	0	0	
n_503_V5_M_126	31	F	Hispanic	No	non-pouchitis		0	0	0	0	
n_503_V6_M_271	31	F	Hispanic	No	non-pouchitis		0	0	0	0	
n_503_V7_M_412	31	F	Hispanic	No	non-pouchitis		0	0	0	0	
