# Supplementary material for: Patient-Specific Bacteroides Genome Variants in Pouchitis
Source: mBio. 2016 Nov 15;7(6):e01713-16. doi: 10.1128/mBio.01713-16 (PMC5111406; doi:10.1128/mBio.01713-16)
Supplement: Table S2 — Summary of linear mixed-effects model results. The coefficients predicted by the linear mixed-effects model for relative abundance of each genus and oligotype in this study are shown. The 97.5% confidence intervals for each coefficient are reported as coef_lwr (coef stands for coefficient, and lwr stands for lower) (2.5%) and coef_upr (upr stands for upper) (97.5%). The uncorrected P value and FDR-corrected P value (adj_pvalue [for adjusted P value]) are reported for each coefficient. Supplemental tables are available at doi:10.6084/m9.figshare.3851478 [file mbo005163055st2.pdf]

Table S2. Summary of linear mixed effects model results

Genera

genus	coefficient	coef_lwr	coef_upr	pvalue	adj_pvalue
Bacteroides	0.272	0.104	0.434	0.002	0.073
Cetobacterium	0.041	-0.057	0.141	0.415	0.981
Fusobacterium	0.010	-0.020	0.040	0.507	0.981
Prevotella	0.009	-0.035	0.053	0.687	0.981
Coprobacillus	0.007	-0.001	0.015	0.076	0.686
Selenomonas	0.005	0.002	0.009	0.004	0.074
Alistipes	0.005	-0.002	0.012	0.182	0.981
Faecalibacterium	0.004	-0.030	0.034	0.794	0.981
Ruminococcaceae	0.003	-0.011	0.017	0.658	0.981
Dialister	0.000	-0.021	0.023	0.981	0.981
Parasutterella	0.000	0.000	0.000	0.888	0.981
Megasphaera	0.000	-0.006	0.006	0.973	0.981
Plesiomonas	0.000	-0.005	0.005	0.914	0.981
Acidaminococcus	-0.001	-0.014	0.013	0.938	0.981
Pseudomonas	-0.001	-0.005	0.003	0.760	0.981
Ruminococcus	-0.001	-0.006	0.005	0.766	0.981
Aquabacterium	-0.001	-0.006	0.005	0.766	0.981
Actinobacillus	-0.001	-0.006	0.004	0.711	0.981
Lachnospira	-0.001	-0.015	0.011	0.880	0.981
Barnesiella	-0.001	-0.007	0.005	0.756	0.981
Sutterella	-0.001	-0.006	0.004	0.655	0.981
Parabacteroides	-0.001	-0.013	0.009	0.840	0.981
Klebsiella	-0.001	-0.006	0.005	0.702	0.981
Coprococcus	-0.001	-0.014	0.011	0.859	0.981
Acinetobacter	-0.002	-0.008	0.005	0.645	0.981
Megamonas	-0.002	-0.013	0.009	0.752	0.981
Subdoligranulum	-0.003	-0.009	0.004	0.455	0.981
Lactococcus	-0.003	-0.010	0.004	0.409	0.981
Dorea	-0.003	-0.010	0.004	0.386	0.981
Clostridium	-0.003	-0.082	0.075	0.936	0.981
Blautia	-0.004	-0.037	0.029	0.810	0.981
Turicibacter	-0.005	-0.057	0.051	0.848	0.981
Epulopiscium	-0.005	-0.022	0.014	0.554	0.981
Lactobacillus	-0.007	-0.032	0.018	0.604	0.981
Peptostreptococcaceae	-0.009	-0.028	0.011	0.393	0.981
Roseburia	-0.011	-0.037	0.016	0.440	0.981
Haemophilus	-0.012	-0.066	0.041	0.648	0.981
Erysipelotrichaceae	-0.016	-0.045	0.014	0.293	0.981
Veillonella	-0.035	-0.097	0.029	0.306	0.981

Enterobacteriaceae	-0.062	-0.139	0.014	0.117	0.820
Streptococcus	-0.079	-0.152	-0.005	0.038	0.536
Lachnospiraceae	-0.084	-0.176	0.011	0.082	0.686

Oligotypes

oligotype	coefficient	coef_lwr	coef_upr	pvalue	adj_pvalue
2373	0.110	0.021	0.199	0.019	0.232
1053	0.044	-0.019	0.108	0.175	0.786
2175	0.044	0.002	0.085	0.044	0.340
1204	0.044	0.013	0.074	0.007	0.124
1938	0.041	-0.006	0.088	0.091	0.584
2425	0.030	-0.045	0.105	0.443	0.941
1992	0.025	0.010	0.041	0.002	0.068
1114	0.023	0.010	0.035	0.001	0.041
1349	0.016	0.003	0.029	0.038	0.317
1775	0.013	0.005	0.021	0.002	0.070
612	0.009	0.003	0.015	0.004	0.094
1727	0.009	0.004	0.013	0.001	0.041
1166	0.008	0.004	0.012	0.000	0.040
216	0.008	-0.028	0.044	0.675	0.976
580	0.008	0.000	0.014	0.037	0.317
284	0.007	-0.003	0.018	0.181	0.786
1235	0.007	0.001	0.012	0.024	0.242
1814	0.006	-0.007	0.020	0.370	0.915
1237	0.006	0.001	0.010	0.012	0.157
1234	0.006	0.001	0.010	0.020	0.242
1489	0.005	0.000	0.010	0.039	0.317
1774	0.005	0.002	0.008	0.003	0.080
1350	0.005	0.002	0.008	0.003	0.082
1643	0.005	0.003	0.007	0.001	0.040
179	0.005	0.002	0.008	0.003	0.080
705	0.005	0.002	0.007	0.001	0.040
1813	0.005	-0.001	0.010	0.116	0.637
674	0.004	0.001	0.006	0.005	0.094
187	0.003	0.001	0.006	0.012	0.157
2021	0.003	0.001	0.005	0.009	0.145
2311	0.003	-0.002	0.008	0.247	0.884
1138	0.003	0.000	0.006	0.051	0.374
2310	0.003	-0.001	0.007	0.179	0.786
1554	0.002	0.001	0.004	0.008	0.130
1206	0.002	0.001	0.003	0.001	0.040
1575	0.002	-0.002	0.006	0.307	0.884
1056	0.002	0.001	0.004	0.007	0.124

2015	0.002	0.001	0.004	0.011	0.157
1236	0.002	0.000	0.004	0.022	0.242
1422	0.002	-0.009	0.011	0.712	0.976
1140	0.002	0.000	0.004	0.045	0.341
2019	0.002	0.000	0.003	0.011	0.157
2316	0.002	-0.003	0.006	0.462	0.941
1142	0.002	0.000	0.003	0.066	0.449
1995	0.001	0.001	0.002	0.001	0.053
1233	0.001	0.000	0.003	0.059	0.416
2016	0.001	0.000	0.003	0.052	0.374
1333	0.001	-0.015	0.019	0.885	0.976
579	0.001	0.000	0.003	0.126	0.667
1116	0.001	0.001	0.002	0.000	0.018
180	0.001	-0.001	0.004	0.376	0.915
393	0.001	0.000	0.002	0.024	0.242
1488	0.001	0.000	0.002	0.037	0.317
876	0.001	-0.004	0.006	0.708	0.976
1208	0.001	0.000	0.002	0.140	0.728
1730	0.001	-0.002	0.003	0.463	0.941
497	0.001	-0.003	0.006	0.722	0.976
1332	0.001	-0.001	0.002	0.280	0.884
2334	0.001	-0.001	0.002	0.451	0.941
1232	0.001	-0.001	0.002	0.297	0.884
1922	0.001	0.000	0.001	0.008	0.130
2426	0.001	0.000	0.002	0.316	0.884
745	0.001	-0.003	0.004	0.760	0.976
2374	0.000	0.000	0.001	0.027	0.264
1464	0.000	-0.003	0.004	0.795	0.976
1577	0.000	0.000	0.001	0.229	0.884
875	0.000	-0.002	0.003	0.751	0.976
1231	0.000	-0.001	0.002	0.527	0.976
1698	0.000	-0.005	0.006	0.885	0.976
58	0.000	0.000	0.001	0.339	0.884
1425	0.000	-0.010	0.011	0.961	0.991
1429	0.000	-0.004	0.005	0.933	0.990
178	0.000	-0.002	0.002	0.912	0.985
1757	0.000	-0.004	0.004	0.963	0.991
283	0.000	-0.001	0.001	0.788	0.976
323	0.000	0.000	0.000	0.590	0.976
1941	0.000	0.000	0.000	0.598	0.976
1148	0.000	0.000	0.000	0.875	0.976
1556	0.000	-0.001	0.001	0.956	0.991

1137	0.000	0.000	0.000	0.950	0.991
2152	0.000	0.000	0.000	0.950	0.991
1630	0.000	-0.001	0.001	0.993	0.993
1961	0.000	0.000	0.000	0.979	0.993
1958	0.000	0.000	0.000	0.972	0.993
2244	0.000	-0.003	0.003	0.993	0.993
1175	0.000	0.000	0.000	0.922	0.986
802	0.000	0.000	0.000	0.939	0.990
2140	0.000	-0.001	0.001	0.958	0.991
930	0.000	0.000	0.000	0.821	0.976
1019	0.000	-0.002	0.002	0.976	0.993
1678	0.000	0.000	0.000	0.851	0.976
2137	0.000	0.000	0.000	0.855	0.976
522	0.000	-0.001	0.001	0.946	0.991
1957	0.000	-0.006	0.006	0.989	0.993
2141	0.000	0.000	0.000	0.839	0.976
395	0.000	-0.001	0.001	0.938	0.990
1507	0.000	-0.001	0.001	0.879	0.976
1041	0.000	-0.002	0.001	0.937	0.990
1021	0.000	-0.001	0.000	0.791	0.976
1928	0.000	-0.001	0.000	0.798	0.976
1052	0.000	0.000	0.000	0.724	0.976
554	0.000	-0.006	0.006	0.983	0.993
613	0.000	-0.001	0.001	0.836	0.976
1268	0.000	-0.001	0.000	0.766	0.976
2424	0.000	-0.001	0.001	0.835	0.976
2448	0.000	0.000	0.000	0.431	0.938
1735	0.000	-0.001	0.001	0.854	0.976
672	0.000	-0.001	0.001	0.862	0.976
2428	0.000	-0.001	0.001	0.801	0.976
1668	0.000	-0.001	0.000	0.694	0.976
2337	0.000	0.000	0.000	0.610	0.976
842	0.000	-0.002	0.002	0.922	0.986
2360	0.000	-0.001	0.000	0.739	0.976
1664	0.000	-0.001	0.001	0.740	0.976
2194	0.000	-0.001	0.000	0.650	0.976
359	0.000	-0.001	0.001	0.830	0.976
946	0.000	-0.001	0.001	0.797	0.976
2230	0.000	-0.001	0.001	0.858	0.976
2453	0.000	0.000	0.000	0.458	0.941
2179	0.000	0.000	0.000	0.433	0.938
1424	0.000	-0.001	0.001	0.762	0.976

1960	0.000	-0.006	0.005	0.966	0.991
502	0.000	-0.001	0.001	0.747	0.976
2427	0.000	-0.001	0.001	0.807	0.976
1273	0.000	-0.001	0.000	0.631	0.976
2405	0.000	-0.001	0.001	0.814	0.976
2206	0.000	-0.002	0.002	0.901	0.979
1646	0.000	-0.001	0.000	0.626	0.976
2485	0.000	0.000	0.000	0.372	0.915
2314	0.000	-0.001	0.001	0.807	0.976
2094	0.000	-0.001	0.000	0.548	0.976
1557	0.000	0.000	0.000	0.441	0.941
1090	0.000	-0.002	0.001	0.849	0.976
1849	0.000	-0.001	0.001	0.765	0.976
1576	0.000	-0.001	0.001	0.737	0.976
1475	0.000	0.000	0.000	0.031	0.293
2255	0.000	-0.001	0.000	0.327	0.884
1666	0.000	-0.001	0.001	0.704	0.976
36	0.000	-0.003	0.002	0.892	0.976
2361	0.000	-0.001	0.000	0.275	0.884
1898	0.000	-0.001	0.000	0.259	0.884
2450	0.000	0.000	0.000	0.183	0.786
1205	0.000	-0.002	0.001	0.812	0.976
1521	0.000	0.000	0.000	0.062	0.428
2139	0.000	-0.001	0.001	0.673	0.976
925	0.000	-0.002	0.002	0.841	0.976
2079	0.000	0.000	0.000	0.162	0.786
929	0.000	-0.001	0.000	0.543	0.976
1508	0.000	-0.003	0.002	0.859	0.976
739	0.000	-0.004	0.003	0.891	0.976
2207	0.000	-0.002	0.001	0.727	0.976
1645	0.000	-0.001	0.001	0.628	0.976
2275	0.000	-0.001	0.000	0.420	0.938
2358	0.000	-0.001	0.000	0.384	0.915
1679	0.000	-0.001	0.000	0.376	0.915
190	0.000	-0.003	0.002	0.827	0.976
1427	0.000	-0.001	0.001	0.620	0.976
1894	0.000	-0.001	0.000	0.423	0.938
1930	0.000	-0.002	0.002	0.790	0.976
1892	0.000	-0.002	0.001	0.734	0.976
1604	0.000	-0.001	0.001	0.607	0.976
1321	0.000	-0.001	0.001	0.575	0.976
1289	0.000	-0.001	0.000	0.177	0.786

1412	0.000	-0.001	0.000	0.309	0.884
286	0.000	-0.003	0.003	0.852	0.976
2196	0.000	-0.001	0.000	0.297	0.884
1295	0.000	-0.001	0.000	0.189	0.796
948	0.000	-0.003	0.002	0.828	0.976
2251	0.000	-0.004	0.003	0.877	0.976
2289	0.000	-0.001	0.000	0.305	0.884
754	0.000	-0.001	0.000	0.324	0.884
1834	0.000	-0.005	0.005	0.904	0.979
2113	0.000	-0.003	0.002	0.812	0.976
1812	0.000	-0.004	0.003	0.846	0.976
862	0.000	-0.001	0.001	0.462	0.941
445	0.000	-0.002	0.002	0.749	0.976
590	0.000	-0.001	0.000	0.304	0.884
2466	0.000	-0.001	0.000	0.022	0.242
2385	0.000	-0.001	0.000	0.043	0.340
1286	0.000	-0.001	0.000	0.180	0.786
2362	0.000	-0.001	0.000	0.227	0.884
1040	0.000	-0.082	0.074	0.992	0.993
795	0.000	-0.001	0.001	0.540	0.976
1811	0.000	-0.005	0.004	0.852	0.976
983	0.000	-0.001	0.000	0.305	0.884
2096	0.000	-0.001	0.000	0.092	0.584
257	0.000	-0.002	0.001	0.474	0.951
675	0.000	-0.004	0.002	0.768	0.976
2246	0.000	-0.002	0.001	0.538	0.976
465	0.000	-0.001	0.000	0.121	0.651
632	0.000	-0.002	0.001	0.596	0.976
1883	0.000	-0.002	0.001	0.360	0.915
960	0.000	-0.005	0.004	0.845	0.976
980	-0.001	-0.001	0.001	0.317	0.884
591	-0.001	-0.001	0.001	0.304	0.884
821	-0.001	-0.002	0.001	0.502	0.972
1633	-0.001	-0.002	0.001	0.520	0.976
2384	-0.001	-0.002	0.001	0.340	0.884
1893	-0.001	-0.002	0.001	0.291	0.884
1573	-0.001	-0.007	0.006	0.848	0.976
1069	-0.001	-0.003	0.002	0.641	0.976
473	-0.001	-0.001	0.000	0.103	0.587
1523	-0.001	-0.001	0.000	0.034	0.311
1603	-0.001	-0.003	0.002	0.597	0.976
1927	-0.001	-0.010	0.007	0.868	0.976

1641	-0.001	-0.004	0.002	0.655	0.976
2335	-0.001	-0.002	0.000	0.246	0.884
2249	-0.001	-0.002	0.001	0.262	0.884
1381	-0.001	-0.002	0.000	0.114	0.635
2336	-0.001	-0.002	0.001	0.312	0.884
2469	-0.001	-0.002	0.001	0.284	0.884
28	-0.001	-0.007	0.005	0.809	0.976
1665	-0.001	-0.010	0.009	0.874	0.976
1675	-0.001	-0.004	0.002	0.591	0.976
1923	-0.001	-0.002	0.001	0.249	0.884
1632	-0.001	-0.004	0.003	0.633	0.976
1754	-0.001	-0.002	0.000	0.088	0.584
44	-0.001	-0.003	0.001	0.361	0.915
2208	-0.001	-0.003	0.001	0.354	0.914
1716	-0.001	-0.004	0.002	0.519	0.976
240	-0.001	-0.007	0.005	0.765	0.976
2153	-0.001	-0.002	0.001	0.216	0.884
2387	-0.001	-0.003	0.001	0.368	0.915
478	-0.001	-0.002	0.000	0.101	0.587
1676	-0.001	-0.004	0.002	0.519	0.976
1476	-0.001	-0.003	0.001	0.218	0.884
52	-0.001	-0.005	0.003	0.606	0.976
355	-0.001	-0.011	0.008	0.821	0.976
751	-0.001	-0.005	0.003	0.601	0.976
1376	-0.001	-0.002	0.000	0.099	0.587
1674	-0.001	-0.009	0.006	0.768	0.976
1375	-0.001	-0.002	0.000	0.094	0.584
2156	-0.001	-0.004	0.002	0.406	0.938
1837	-0.001	-0.006	0.003	0.606	0.976
2288	-0.001	-0.007	0.004	0.657	0.976
2158	-0.001	-0.003	0.001	0.253	0.884
470	-0.001	-0.003	0.001	0.150	0.765
1380	-0.001	-0.003	0.000	0.099	0.587
752	-0.001	-0.005	0.002	0.499	0.972
1463	-0.001	-0.012	0.008	0.780	0.976
947	-0.001	-0.011	0.008	0.787	0.976
389	-0.001	-0.011	0.010	0.830	0.976
1838	-0.001	-0.005	0.003	0.495	0.971
840	-0.002	-0.006	0.003	0.533	0.976
1165	-0.002	-0.010	0.007	0.731	0.976
2449	-0.002	-0.004	0.001	0.178	0.786
540	-0.002	-0.005	0.002	0.336	0.884

2048	-0.002	-0.007	0.004	0.552	0.976
1648	-0.002	-0.007	0.003	0.525	0.976
469	-0.002	-0.006	0.002	0.410	0.938
1789	-0.002	-0.010	0.006	0.679	0.976
1410	-0.002	-0.005	0.002	0.320	0.884
1287	-0.002	-0.006	0.003	0.476	0.951
1470	-0.002	-0.005	0.002	0.336	0.884
392	-0.002	-0.013	0.009	0.746	0.976
595	-0.002	-0.006	0.002	0.315	0.884
750	-0.002	-0.011	0.005	0.651	0.976
977	-0.002	-0.005	0.002	0.295	0.884
701	-0.002	-0.012	0.008	0.675	0.976
1318	-0.002	-0.007	0.002	0.283	0.884
797	-0.002	-0.008	0.003	0.407	0.938
963	-0.002	-0.011	0.007	0.598	0.976
553	-0.002	-0.019	0.013	0.772	0.976
2204	-0.003	-0.009	0.004	0.465	0.941
1423	-0.003	-0.053	0.047	0.921	0.986
538	-0.003	-0.011	0.006	0.536	0.976
255	-0.003	-0.009	0.004	0.415	0.938
820	-0.003	-0.043	0.036	0.895	0.976
1524	-0.003	-0.010	0.005	0.463	0.941
1853	-0.003	-0.007	0.001	0.239	0.884
709	-0.003	-0.010	0.004	0.429	0.938
1070	-0.003	-0.013	0.007	0.573	0.976
2386	-0.003	-0.011	0.006	0.490	0.967
1855	-0.003	-0.007	0.001	0.155	0.765
1322	-0.003	-0.008	0.002	0.239	0.884
1526	-0.003	-0.009	0.003	0.317	0.884
2114	-0.003	-0.032	0.023	0.809	0.976
630	-0.003	-0.014	0.011	0.595	0.976
800	-0.004	-0.010	0.003	0.283	0.884
536	-0.004	-0.028	0.020	0.763	0.976
943	-0.004	-0.017	0.009	0.579	0.976
2274	-0.004	-0.011	0.003	0.268	0.884
1088	-0.004	-0.047	0.037	0.852	0.976
1851	-0.004	-0.011	0.002	0.224	0.884
1522	-0.004	-0.008	0.000	0.024	0.242
710	-0.004	-0.065	0.059	0.889	0.976
1790	-0.005	-0.022	0.009	0.592	0.976
1736	-0.005	-0.024	0.009	0.565	0.976
24	-0.005	-0.016	0.007	0.382	0.915

1753	-0.006	-0.027	0.016	0.619	0.976
1267	-0.006	-0.041	-0.001	0.740	0.976
2077	-0.006	-0.015	0.002	0.154	0.765
1885	-0.006	-0.050	0.038	0.779	0.976
2404	-0.008	-0.028	0.012	0.459	0.941
1068	-0.008	-0.022	0.009	0.332	0.884
2231	-0.008	-0.028	0.012	0.424	0.938
1469	-0.008	-0.024	0.012	0.379	0.915
1835	-0.009	-0.045	0.031	0.666	0.976
238	-0.009	-0.055	0.037	0.713	0.976
2138	-0.009	-0.029	0.014	0.433	0.938
1836	-0.010	-0.052	0.028	0.640	0.976
2357	-0.012	-0.035	0.013	0.325	0.884
1605	-0.014	-0.057	0.034	0.559	0.976
2203	-0.014	-0.048	0.020	0.417	0.938
589	-0.015	-0.039	0.011	0.243	0.884
796	-0.017	-0.046	0.012	0.255	0.884
2042	-0.020	-0.076	0.035	0.478	0.951
253	-0.023	-0.077	0.030	0.402	0.938
2483	-0.029	-0.074	0.007	0.184	0.786
2093	-0.029	-0.071	0.013	0.168	0.786
2500	-0.032	-0.070	0.007	0.103	0.587
468	-0.034	-0.082	0.019	0.197	0.818
