# Supplementary material for: Patient-Specific Bacteroides Genome Variants in Pouchitis
Source: mBio. 2016 Nov 15;7(6):e01713-16. doi: 10.1128/mBio.01713-16 (PMC5111406; doi:10.1128/mBio.01713-16)
Supplement: Table S3 — Abundant Bacteroides oligotypes and MAGs isolated from pouchitis patients during inflammation. The letters a to e indicate unique Bacteroides MED oligotypes detected during inflammation with “na” indicating samples not available from visits during inflammation. Taxonomy of the oligotype is based on similarity to hits in the NCBI RefSeq rRNA database. The table reports the most abundant Bacteroides MAG and the percent recruitment during inflammation and MAG taxonomy assignments based on similarity to genomes contained in the RAST database. Supplemental tables are available at doi:10.6084/m9.figshare.3851478 [file mbo005163055st3.pdf]

Table S3. Abundant *Bacteroides* oligotypes and MAGs isolated from pouchitis patients during inflammation.

patient	days after takedown	Oligotype ID	Oligotype taxonomy	Oligotype % abundance	MAG	MAG % read recruitment
p-204	434	b	*B.fragilis*	42.6	*B.fragilis*	23.7
p-207	482	b	*B.fragilis*	64.4	*B.fragilis*	25.6
p-214	484	b	*B.fragilis*	70.6	*B.fragilis*	61.6
p-200	282	a	*B.ovatus*	8.2	*B.thetaiotamicron*	16.7
p-200	352	a	*B.ovatus*	29.4	*B.thetaiotamicron*	11.3
p-208	613	d	*B.ovatus*	24.6	*B.ovatus*	49.2
p-219	211	a	*B.ovatus*	8.0	*B.thetaiotamicron*	1.4
p-207	719	c	*B.vulgatus*	73.2	*B.vulgatus*	23.6
p-202	301	e	*B.vulgatus*	0.44	*B.vulgatus*	0.19
p-206	*na*	*na*	*na*	*na*	*B.vulgatus*	< 0.1
p-500	*na*	*na*	*na*	*na*	*na*	*na*
p-502	*na*	*na*	*na*	*na*	*na*	*na*
p-218	301	undetected	undetected	*na*	undetected	undetected
