# Supplementary material for: Patient-Specific Bacteroides Genome Variants in Pouchitis
Source: mBio. 2016 Nov 15;7(6):e01713-16. doi: 10.1128/mBio.01713-16 (PMC5111406; doi:10.1128/mBio.01713-16)
Supplement: Table S4 — Summary of MAGs recovered from each patient. The summary includes the patient ID, unique MAG ID, taxonomy, summary of the MAG size, common assembly statistics, and completion/contamination based on three bacterial single-copy core gene references. Taxonomy is based on the most common hit of the MAG contigs to genomes in the RAST database. Supplemental tables are available at doi:10.6084/m9.figshare.3851478. The supplemental material at doi:10.6084/m9.figshare.3851364 includes anvi'o profiles to visualize and re-analyze metagenomes for each patient, and MAGs reported in the manuscript. [file mbo005163055st4.pdf]

Table S4. Summary of MAGs recovered from each patient.

patient	MAG	RAST - Taxonomy	Num Contigs	N50	GC Content	Total Size (Mb)	Completion	Redundant
p-200	p-200-10	*Bacteroides thetaiotaomicron*	83	121,198	42.58%	5.95	68.76%	2.80%
p-200	p-200-11-1	*Bacteroides ovatus*	97	94189	41.90%	6.15	54.35%	4.05%
p-200	p-200-11-2	*Bacteroides stercoris*	64	72970	46.20%	3.33	54.76%	3.87%
p-200	p-200-13	*Bacteroides uniformis*	34	120,000	47.13%	3.10	40.03%	0.45%
p-200	p-200-14	*Bacteroides stercoris*	34	107,755	47.29%	2.33	39.41%	0.63%
p-200	p-200-23	*Bacteroides massiliensis*	80	51,981	41.73%	2.71	89.26%	3.75%
p-202	p-202-30	*Bacteroides thetaiotaomicron*	193	36,369	42.51%	5.12	42.94%	9.15%
p-202	p-202-31	*Bacteroides vulgatus*	81	93,227	42.10%	4.85	56.84%	10.92%
p-204	p-204-37	*Bacteroides thetaiotaomicron*	247	14,617	41.18%	2.94	15.88%	1.26%
p-204	p-204-4	*Bacteroides vulgatus*	85	117,576	41.77%	5.06	98.26%	8.42%
p-204	p-204-5	*Bacteroides stercoris*	104	59,427	45.64%	4.11	96.35%	9.38%
p-204	p-204-7	*Bacteroides fragilis*	62	112,911	42.82%	4.50	93.05%	9.91%
p-206	p-206-22	*Bacteroides massiliensis*	77	58,568	42.39%	2.83	94.79%	6.21%
p-207	p-207-31	*Bacteroides thetaiotaomicron*	24	145,272	40.86%	2.86	22.52%	3.23%
p-207	p-207-32	*Bacteroides vulgatus*	128	51,034	42.16%	4.81	97.48%	10.04%
p-207	p-207-33	*Bacteroides fragilis*	46	177,543	43.49%	4.64	93.46%	8.01%
p-208	p-208-28	*Bacteroides vulgatus*	80	83,188	42.10%	4.39	94.49%	6.87%
p-208	p-208-29	*Bacteroides thetaiotaomicron*	64	190,201	41.63%	6.25	53.72%	8.31%
n-210	n-210-1	*Bacteroides vulgatus*	81	120,936	41.75%	5.19	98.29%	6.62%
n-210	n-210-15	*Bacteroides thetaiotaomicron*	319	17,594	42.99%	4.51	15.78%	1.26%
n-210	n-210-2	*Bacteroides thetaiotaomicron*	243	21,714	41.70%	3.93	21.39%	0.81%
n-210	n-210-23	*Bacteroides nordii*	252	13,618	40.16%	2.91	9.95%	4.60%
n-210	n-210-3	*Bacteroides caccae*	158	47,127	42.02%	4.94	60.98%	3.56%
n-210	n-210-4	*Bacteroides thetaiotaomicron*	248	23,386	41.73%	4.42	22.15%	1.26%
n-210	n-210-6	*Bacteroides fragilis*	85	103,820	43.04%	4.80	53.18%	5.64%
n-211	n-211-36	*Bacteroides uniformis*	109	47,046	47.51%	3.90	51.52%	8.85%
n-212	n-212-31	*Bacteroides fragilis*	47	164,617	43.31%	4.74	97.42%	7.53%
n-212	n-212-6	*Bacteroides capillosus*	162	24,713	62.15%	3.03	92.74%	9.84%
n-213	n-213-19	*Bacteroides vulgatus*	35	112,247	40.43%	2.60	29.24%	5.91%
n-213	n-213-31	*Bacteroides fragilis*	161	21,484	44.89%	2.63	35.07%	0.84%
n-213	n-213-32	*Bacteroides vulgatus*	41	93,067	43.29%	2.49	59.32%	1.32%
p-214	p-214-19	*Bacteroides fragilis*	60	142,762	43.29%	4.78	97.18%	7.23%
p-215	p-215-16	*Bacteroides vulgatus*	95	90,928	41.40%	5.18	98.26%	8.96%
p-215	p-215-17	*Bacteroides thetaiotaomicron*	368	17,358	41.79%	5.23	30.61%	4.65%
p-215	p-215-18-1	*Bacteroides capillosus*	221	12437	62.40%	2.00	71.55%	1.83%
p-215	p-215-5	*Bacteroides fragilis*	77	125,750	43.17%	4.96	96.34%	8.01%
n-216	n-216-1	*Bacteroides fragilis*	84	82,997	43.36%	4.65	59.34%	7.77%
n-216	n-216-2	*Bacteroides vulgatus*	53	123,233	41.93%	4.59	65.44%	7.11%
n-216	n-216-3	*Bacteroides thetaiotaomicron*	218	10,276	44.11%	2.14	14.97%	0.30%
n-217	n-217-6	*Bacteroides uniformis*	54	100,395	46.90%	4.33	98.29%	4.74%
p-219	p-219-1	*Bacteroides thetaiotaomicron*	93	112,326	41.89%	6.80	97.12%	7.23%
n-427	n-427-1	*Bacteroides fragilis*	192	32,630	43.41%	4.77	63.18%	3.47%
n-427	n-427-11	*Bacteroides thetaiotaomicron*	180	51,518	41.66%	6.12	71.99%	4.41%
n-427	n-427-24	*Bacteroides vulgatus*	173	43,251	42.39%	4.91	52.93%	5.44%
n-427	n-427-B.uniformis	*Bacteroides uniformis*	93	73,838	46.74%	4.61	67.10%	5.55%
p-500	p-500-12	*Bacteroides thetaiotaomicron*	259	32,996	41.65%	5.86	39.92%	3.06%
p-500	p-500-14	*Bacteroides uniformis*	126	50,723	46.26%	4.34	77.02%	2.02%
p-500	p-500-15	*Bacteroides thetaiotaomicron*	205	44,405	42.94%	5.47	45.93%	4.96%
p-500	p-500-2	*Bacteroides vulgatus*	209	30,153	41.67%	4.39	91.04%	1.84%
p-500	p-500-21	*Bacteroides intestinalis*	242	41,284	43.25%	6.58	58.11%	4.19%
p-500	p-500-27-1	*Bacteroides vulgatus*	225	15436	47.10%	3.09	44.56%	6.88%
p-500	p-500-4	*Bacteroides thetaiotaomicron*	156	15,820	43.54%	2.04	15.24%	0.00%
p-500	p-500-7	*Bacteroides thetaiotaomicron*	187	17,584	42.55%	2.70	19.17%	2.17%
p-502	p-502-26	*Bacteroides capillosus*	217	9,665	61.47%	2.02	35.95%	1.39%
p-502	p-502-28	*Bacteroides uniformis*	158	43,122	46.05%	4.37	91.90%	5.51%
p-502	p-502-39	*Bacteroides vulgatus*	321	11,699	41.95%	3.42	56.81%	1.66%
p-502	p-502-40	*Bacteroides massiliensis*	102	27,621	42.35%	2.09	50.35%	0.68%
p-502	p-502-Yjeta_01	*Bacteroides thetaiotaomicron*	215	35,585	42.56%	5.69	51.66%	3.42%
p-502	p-502-Yjeta_02	*Bacteroides thetaiotaomicron*	286	16,652	41.62%	3.83	10.92%	0.23%
p-502	p-502-Yjeta_03	*Bacteroides thetaiotaomicron*	290	21,113	41.32%	4.56	10.94%	0.00%
n-503	n-503-11	*Bacteroides capillosus*	170	13,380	63.81%	2.08	82.89%	4.78%

n-503	n-503-25	*Bacteroides thetaiotaomicron*	57	118,812	43.69%	4.32	36.94%	0.63%
n-503	n-503-37	*Bacteroides vulgatus*	86	125,797	42.00%	5.40	49.46%	6.24%
n-210	n-210-5	*Acidaminococcus sp.*	56	68,215	50.06%	2.17	92.76%	5.07%
n-503	n-503-16	*Acidaminococcus sp.*	52	61,365	50.34%	2.04	98.65%	4.45%
n-210	n-210-44	*Akkermansia muciniphila*	43	107,783	56.64%	3.11	98.51%	2.75%
n-503	n-503-9	*Akkermansia muciniphila*	33	97,289	55.88%	2.51	96.25%	2.35%
n-210	n-210-11	*Alistipes onderdonkii*	45	120,182	58.02%	3.05	97.88%	3.57%
n-210	n-210-12	*Alistipes shahii*	76	62,763	57.88%	3.10	97.88%	3.98%
n-210	n-210-3	*Bacteroides caccae*	158	47,127	42.02%	4.94	60.98%	3.56%
n-212	n-212-6	*Bacteroides capillosus*	162	24,713	62.15%	3.03	92.74%	9.84%
n-503	n-503-11	*Bacteroides capillosus*	170	13,380	63.81%	2.08	82.89%	4.78%
p-502	p-502-26	*Bacteroides capillosus*	217	9,665	61.47%	2.02	35.95%	1.39%
p-500	p-500-23	*Barnesiella intestinihominis*	63	101,836	43.28%	3.18	97.71%	4.71%
n-216	n-216-20	*Bifidobacterium adolescentis*	141	17,101	59.54%	1.97	64.71%	6.09%
n-211	n-211-1	*Bifidobacterium adolescentis*	46	69,706	59.70%	2.19	94.66%	5.67%
p-204	p-204-26	*Bifidobacterium adolescentis*	26	101,503	60.42%	1.69	88.18%	5.43%
p-207	p-207-5	*Bifidobacterium adolescentis*	24	122,133	59.57%	2.01	94.37%	4.35%
p-202	p-202-6	*Bifidobacterium animalis*	51	69,959	61.04%	1.89	94.90%	7.04%
n-210	n-210-26	*Bifidobacterium bifidum*	110	23,479	62.82%	1.95	75.43%	3.55%
p-202	p-202-5	*Bifidobacterium breve*	29	130,494	58.51%	2.32	96.58%	6.51%
p-206	p-206-1	*Bifidobacterium dentium*	98	34,856	59.13%	2.19	60.64%	4.25%
n-217	n-217-3	*Bifidobacterium longum*	68	64,330	60.12%	2.42	96.18%	7.54%
n-213	n-213-2	*Bifidobacterium longum*	50	65,705	60.28%	2.45	96.52%	6.50%
n-212	n-212-8	*Bifidobacterium longum*	87	35,876	60.78%	2.08	88.02%	4.41%
n-423	n-423-13	*Bifidobacterium longum*	37	83,759	60.05%	2.29	95.10%	2.84%
p-204	p-204-44	*Bifidobacterium longum*	106	18,606	59.81%	1.56	65.30%	2.80%
p-207	p-207-4	*Bifidobacterium longum*	37	100,510	60.06%	2.06	91.79%	1.85%
p-215	p-215-1	*Bifidobacterium pseudocatenulatum*	39	80,891	56.42%	2.07	94.66%	6.21%
n-217	n-217-1	*Bifidobacterium pseudocatenulatum*	21	144,194	56.18%	1.99	97.03%	4.38%
n-210	n-210-57	*Bifidobacterium pseudocatenulatum*	82	25,554	55.73%	1.55	39.62%	2.84%
n-210	n-210-41	*Bilophila wadsworthia*	275	15,513	59.00%	3.64	82.78%	4.69%
n-503	n-503-10	*Bilophila wadsworthia*	207	22,375	60.20%	3.66	94.20%	3.98%
p-204	p-204-38	*Blautia hansenii*	134	21,522	38.83%	2.17	45.44%	8.78%
p-206	p-206-31	*Blautia hansenii*	84	28,638	41.29%	1.91	31.85%	8.23%
n-427	n-427-13	*Blautia hansenii*	107	37,298	38.57%	2.70	58.76%	7.30%
p-208	p-208-37	*Blautia hansenii*	120	21,602	39.01%	2.03	49.04%	7.24%
n-427	n-427-2	*Blautia hansenii*	145	25,047	43.83%	2.86	52.79%	6.22%
p-208	p-208-33	*Blautia hansenii*	134	16,918	44.92%	1.79	37.00%	5.56%
p-206	p-206-32	*Blautia hansenii*	127	20,561	41.65%	2.11	33.77%	4.96%
p-200	p-200-25	*Blautia hansenii*	162	23,738	41.10%	2.83	37.84%	4.81%
p-500	p-500-13	*Burkholderiales bacterium*	74	68,919	48.29%	2.77	98.51%	5.41%
n-211	n-211-5	*Butyricicoccus pullicaecorum*	111	24,708	55.56%	1.93	62.96%	5.96%
n-210	n-210-18	*Butyricicoccus pullicaecorum*	117	25,466	54.99%	2.23	91.87%	3.75%
p-215	p-215-6	*Clostridiaceae bacterium*	70	54,293	36.12%	2.49	92.99%	10.20%
p-208	p-208-16	*Clostridiales bacterium*	270	20,370	51.54%	4.25	33.35%	8.01%
n-503	n-503-8	*Clostridiales bacterium*	183	33,892	56.85%	4.90	41.31%	5.53%
n-212	n-212-20	*Clostridium bartlettii*	86	40,372	29.01%	2.06	37.68%	9.01%
p-218	p-218-1	*Clostridium bartlettii*	146	11,269	28.59%	1.55	80.53%	6.45%
n-503	n-503-39	*Clostridium bartlettii*	228	16,958	28.52%	3.26	44.83%	5.44%
p-200	p-200-8	*Clostridium bartlettii*	168	18,690	28.26%	2.57	77.27%	4.48%
n-423	n-423-8	*Clostridium bartlettii*	190	10,443	29.64%	1.87	17.01%	1.66%
n-217	n-217-9	*Clostridium beijerincki*	129	46,864	28.52%	4.34	82.94%	5.93%
n-503	n-503-15	*Clostridium beijerincki*	122	39,549	28.67%	3.49	83.66%	5.57%
n-423	n-423-10	*Clostridium beijerincki*	207	23,988	28.43%	3.69	33.51%	2.73%
n-216	n-216-17	*Clostridium bolteae*	365	15,465	50.09%	4.61	32.11%	10.28%
p-207	p-207-34	*Clostridium bolteae*	207	12,791	51.22%	2.36	35.51%	8.90%
p-502	p-502-20	*Clostridium bolteae*	264	20,600	50.48%	4.30	40.99%	6.50%
p-500	p-500-29	*Clostridium bolteae*	311	19,375	48.74%	4.94	49.89%	5.89%
n-209	n-209-20	*Clostridium bolteae*	166	33,127	50.62%	3.87	55.11%	4.09%
n-212	n-212-22	*Clostridium bolteae*	237	9,670	50.74%	2.24	25.44%	2.75%
n-503	n-503-20	*Clostridium bolteae*	211	24,021	51.09%	3.71	31.49%	1.66%
p-202	p-202-19	*Clostridium bolteae*	217	9,456	50.70%	1.97	11.43%	0.72%
n-210	n-210-45	*Clostridium bolteae*	204	10,201	48.42%	1.98	8.90%	0.00%

n-427	n-427-20	*Clostridium bolteae*	249	9,227	49.76%	2.23	33.95%	0.00%
p-206	p-206-10	*Clostridium celatum*	88	43,677	27.95%	2.54	29.93%	10.15%
p-215	p-215-8	*Clostridium celatum*	59	70,737	28.47%	2.06	67.85%	8.60%
n-211	n-211-11	*Clostridium celatum*	191	9,162	27.93%	1.75	46.07%	7.17%
p-200	p-200-7	*Clostridium celatum*	121	40,449	27.65%	3.12	44.57%	6.40%
p-502	p-502-2	*Clostridium celatum*	187	16,195	27.94%	2.65	96.49%	5.56%
n-423	n-423-21	*Clostridium celatum*	87	29,795	27.55%	2.01	24.75%	3.78%
n-503	n-503-18	*Clostridium hathewayi*	322	15,027	49.85%	4.19	34.76%	1.62%
p-218	p-218-5	*Clostridium perfringens*	198	15,104	28.12%	2.50	54.94%	8.00%
p-219	p-219-5	*Clostridium perfringens*	193	13,134	28.40%	2.17	88.43%	7.17%
p-200	p-200-6	*Clostridium perfringens*	178	10,741	28.38%	1.81	73.71%	2.11%
n-216	n-216-19	*Clostridium ramosum*	84	52,943	31.13%	2.75	52.65%	10.21%
p-206	p-206-8	*Clostridium ramosum*	113	32,450	31.28%	2.77	80.43%	9.97%
n-217	n-217-8	*Clostridium ramosum*	45	107,250	31.29%	3.12	98.69%	6.10%
p-500	p-500-11	*Clostridium ramosum*	80	68,845	31.43%	3.53	98.69%	6.10%
p-502	p-502-7	*Clostridium ramosum*	62	83,445	31.13%	3.16	48.19%	5.37%
n-423	n-423-3	*Clostridium ramosum*	248	9,014	31.49%	2.21	88.23%	4.56%
n-503	n-503-13	*Clostridium ramosum*	82	56,691	31.23%	2.94	49.15%	4.23%
p-200	p-200-2	*Clostridium ramosum*	68	60,085	31.30%	2.83	50.36%	3.87%
p-214	p-214-16	*Clostridium scindens*	179	15,463	46.30%	2.54	34.23%	7.48%
n-210	n-210-61	*Clostridium scindens*	191	20,512	47.60%	3.27	44.25%	3.70%
p-202	p-202-16	*Clostridium sp.*	49	69,554	37.42%	2.23	90.71%	10.75%
n-211	n-211-26	*Clostridium sp.*	68	46,880	37.15%	2.50	90.42%	10.15%
p-214	p-214-12	*Clostridium sp.*	142	19,447	37.33%	2.26	45.75%	7.23%
p-208	p-208-39	*Clostridium sp.*	154	16,139	37.41%	2.02	51.18%	6.15%
p-207	p-207-14	*Clostridium sp.*	185	14,337	36.89%	2.17	34.51%	5.26%
n-423	n-423-30	*Clostridium sp.*	151	18,537	36.91%	2.29	36.98%	4.81%
p-215	p-215-10	*Clostridium sp.*	148	14,907	37.25%	1.93	51.59%	4.49%
n-423	n-423-26	*Clostridium sp.*	143	20,348	36.74%	2.35	40.61%	4.09%
p-200	p-200-39	*Clostridium sp.*	131	16,834	50.50%	1.89	32.78%	2.80%
p-200	p-200-24	*Clostridium sp.*	160	11,104	37.44%	1.68	29.02%	2.24%
n-210	n-210-30	*Clostridium sp.*	72	31,898	36.99%	1.62	59.18%	0.80%
n-211	n-211-13	*Clostridium spiroforme*	62	40,507	27.91%	1.80	62.61%	10.37%
n-209	n-209-9	*Clostridium spiroforme*	79	34,070	28.13%	1.95	44.20%	4.00%
p-200	p-200-3	*Clostridium spiroforme*	132	18,649	28.39%	1.87	40.46%	1.88%
p-202	p-202-4	*Clostridium symbiosum*	258	12,863	48.52%	3.04	35.75%	6.28%
p-214	p-214-17	*Clostridium symbiosum*	221	19,738	48.60%	3.51	40.53%	6.22%
p-502	p-502-15	*Clostridium symbiosum*	294	17,872	48.00%	4.48	61.38%	4.77%
p-215	p-215-22	*Clostridium symbiosum*	142	15,970	49.87%	1.86	36.97%	3.65%
p-208	p-208-10	*Clostridium symbiosum*	255	9,705	48.31%	2.45	24.68%	2.75%
n-210	n-210-21	*Clostridium symbiosum*	156	20,373	48.07%	2.49	58.57%	2.24%
n-427	n-427-19	*Clostridium symbiosum*	203	7,832	47.64%	1.60	32.10%	1.14%
n-212	n-212-7	*Collinsella aerofaciens*	61	53,202	60.19%	2.05	95.74%	9.61%
p-215	p-215-24	*Collinsella aerofaciens*	62	46,620	60.17%	2.07	91.61%	8.48%
p-206	p-206-2	*Collinsella aerofaciens*	63	53,076	59.86%	2.13	97.90%	7.46%
p-200	p-200-36	*Collinsella aerofaciens*	206	9,754	60.03%	1.88	75.11%	7.17%
n-423	n-423-2	*Collinsella aerofaciens*	45	69,811	59.83%	2.17	98.47%	3.75%
n-210	n-210-8	*Collinsella aerofaciens*	51	78,485	59.97%	2.18	98.47%	3.24%
p-500	p-500-8	*Collinsella stercoris*	38	72,957	62.47%	1.77	97.93%	2.57%
n-209	n-209-11	*Coprobacillus sp.*	65	62,545	31.32%	2.35	97.93%	6.59%
p-214	p-214-14	*Dorea formicigenerans*	154	13,064	41.24%	1.73	17.01%	4.83%
n-503	n-503-21	*Dorea formicigenerans*	77	36,662	41.21%	2.18	60.65%	4.33%
n-210	n-210-51	*Dorea formicigenerans*	149	21,358	40.48%	2.59	53.13%	3.41%
p-204	p-204-30	*Dorea longicatena*	57	58,685	41.61%	2.02	72.49%	7.94%
p-502	p-502-10	*Dorea longicatena*	105	33,397	42.08%	2.38	67.72%	5.75%
p-207	p-207-16	*Enterococcus casseliflavus*	151	13,767	38.35%	1.87	56.10%	8.71%
p-200	p-200-31	*Enterococcus casseliflavus*	190	10,036	39.78%	1.83	33.63%	2.79%
n-212	n-212-16	*Enterococcus faecalis*	160	12,594	37.74%	1.83	54.33%	5.67%
n-211	n-211-22	*Erysipelotrichaceae bacterium*	154	15,458	37.85%	2.02	36.57%	6.02%
n-503	n-503-28	*Erysipelotrichaceae bacterium*	82	77,824	44.25%	3.66	91.48%	4.81%
n-427	n-427-16	*Erysipelotrichaceae bacterium*	258	11,342	44.46%	2.69	74.74%	2.95%
p-200	p-200-20	*Erysipelotrichaceae bacterium*	210	12,771	45.05%	2.40	34.57%	2.35%
n-216	n-216-23	*Escherichia coli*	74	98,128	50.92%	4.48	65.68%	10.69%

n-210	n-210-70	*Escherichia coli*	97	71,236	41.75%	2.55	97.88%	6.95%
p-218	p-218-21	*Escherichia coli*	268	16,406	51.09%	3.52	76.03%	6.69%
p-202	p-202-10	*Escherichia coli*	295	11,470	51.13%	3.09	83.65%	6.51%
n-211	n-211-3	*Escherichia coli*	211	17,758	51.93%	2.99	68.84%	5.14%
n-217	n-217-5	*Escherichia coli*	99	105,990	50.49%	4.91	98.29%	3.93%
p-207	p-207-6	*Escherichia coli*	195	10,480	51.68%	1.92	68.18%	2.64%
n-503	n-503-41	*Escherichia coli*	253	24,701	50.55%	4.50	26.26%	0.85%
p-200	p-200-42	*Escherichia coli*	156	11,138	49.96%	1.72	1.08%	0.18%
p-200	p-200-43	*Escherichia coli*	191	9,162	50.52%	1.65	44.93%	0.00%
p-500	p-500-30	*Escherichia coli*	313	14,310	50.18%	3.89	35.69%	0.00%
n-216	n-216-14	*Eubacterium eligens*	113	31,321	37.63%	2.41	81.39%	10.33%
n-212	n-212-35	*Eubacterium eligens*	135	18,851	35.94%	2.14	41.03%	8.83%
p-208	p-208-23	*Eubacterium eligens*	180	8,794	36.98%	1.52	31.43%	7.53%
n-423	n-423-14	*Eubacterium eligens*	71	62,547	35.96%	2.66	97.03%	7.34%
n-217	n-217-15	*Eubacterium eligens*	64	57,268	36.09%	2.41	68.56%	5.03%
n-211	n-211-16	*Eubacterium eligens*	145	13,737	36.12%	1.75	25.45%	2.15%
n-210	n-210-19	*Eubacterium eligens*	185	17,474	35.44%	2.66	54.85%	1.79%
p-207	p-207-15	*Eubacterium hallii*	149	12,595	37.99%	1.72	40.65%	5.79%
n-210	n-210-58	*Eubacterium hallii*	176	12,360	38.50%	1.99	37.20%	3.14%
n-210	n-210-7	*Eubacterium rectale*	106	44,685	40.89%	3.07	94.90%	8.23%
n-213	n-213-13	*Eubacterium rectale*	64	43,227	42.51%	2.00	44.25%	7.93%
p-202	p-202-35	*Eubacterium rectale*	123	17,969	44.64%	1.91	26.03%	5.79%
n-217	n-217-11	*Eubacterium rectale*	76	47,320	41.82%	2.47	53.53%	5.48%
n-210	n-210-42	*Eubacterium rectale*	128	25,790	44.13%	2.53	86.38%	5.44%
p-200	p-200-12	*Eubacterium rectale*	46	69,723	42.18%	2.21	46.03%	4.63%
n-211	n-211-15	*Eubacterium ventriosum*	119	19,879	33.31%	1.89	57.36%	8.00%
p-202	p-202-7	*Faecalibacterium prausnitzii*	42	88,666	59.21%	1.99	79.28%	9.67%
p-200	p-200-41	*Faecalibacterium prausnitzii*	35	110,127	57.56%	2.47	98.47%	6.17%
n-210	n-210-13	*Faecalibacterium prausnitzii*	112	28,856	56.04%	2.36	51.92%	5.84%
n-210	n-210-9	*Faecalibacterium prausnitzii*	82	48,443	57.64%	2.39	58.33%	5.84%
n-423	n-423-12	*Faecalibacterium prausnitzii*	67	35,820	60.14%	1.78	62.94%	5.26%
p-502	p-502-11	*Faecalibacterium prausnitzii*	121	32,733	56.15%	2.43	61.70%	5.17%
n-212	n-212-18	*Fusobacterium ulcerans*	61	56,140	31.46%	2.18	94.37%	10.04%
p-500	p-500-5	*Fusobacterium ulcerans*	52	60,631	31.45%	2.20	97.33%	4.34%
p-500	p-500-22	*Fusobacterium ulcerans*	160	11,940	29.34%	1.68	87.71%	2.40%
p-207	p-207-21	*Lachnospiraceae bacterium*	60	62,652	42.24%	2.22	61.66%	10.14%
p-208	p-208-42	*Lachnospiraceae bacterium*	49	60,801	42.37%	2.05	66.81%	8.53%
n-216	n-216-8	*Lachnospiraceae bacterium*	105	19,846	42.97%	1.72	43.05%	6.15%
n-212	n-212-41	*Lachnospiraceae bacterium*	36	67,143	42.30%	1.90	71.96%	5.79%
n-427	n-427-6	*Lachnospiraceae bacterium*	87	50,656	41.60%	2.57	84.28%	4.99%
n-503	n-503-23	*Lachnospiraceae bacterium*	59	51,479	41.98%	2.43	58.62%	4.59%
n-210	n-210-28	*Lachnospiraceae bacterium*	78	51,942	41.24%	2.77	62.47%	4.41%
n-211	n-211-31	*Lachnospiraceae bacterium*	116	18,770	42.92%	1.67	45.92%	3.89%
p-500	p-500-24	*Lachnospiraceae bacterium*	137	16,583	42.27%	1.84	42.40%	3.22%
n-503	n-503-5	*Lactobacillus fermentum*	56	48,668	53.12%	1.72	96.11%	3.75%
n-209	n-209-37	*Lactococcus lactis*	79	38,470	35.11%	2.07	93.55%	4.11%
p-500	p-500-6	*Megamonas funiformis*	89	37,097	31.56%	2.26	97.12%	4.96%
n-427	n-427-14	*Megasphaera micronuciformis*	32	92,150	45.56%	1.68	98.87%	4.42%
n-210	n-210-38	*Odoribacter splanchnicus*	192	28,202	43.29%	4.04	75.73%	2.02%
n-216	n-216-5	*Parabacteroides distasonis*	42	167,136	45.35%	4.39	96.94%	9.62%
p-215	p-215-4	*Parabacteroides distasonis*	54	148,025	44.95%	4.93	98.26%	8.90%
p-204	p-204-17	*Parabacteroides distasonis*	78	92,814	45.10%	4.45	96.58%	8.30%
n-211	n-211-34	*Parabacteroides distasonis*	108	56,408	45.31%	4.40	81.33%	8.06%
p-500	p-500-16	*Parabacteroides distasonis*	93	76,571	45.21%	4.99	98.69%	5.83%
p-500	p-500-9	*Parabacteroides merdae*	117	59,237	45.11%	4.53	98.69%	5.27%
n-211	n-211-35	*Parabacteroides merdae*	143	23,730	47.35%	2.51	25.45%	1.02%
n-210	n-210-10	*Phascolarctobacterium sp.*	29	174,921	43.59%	2.41	98.87%	5.30%
p-202	p-202-9	*Prevotella buccae*	113	39,196	52.18%	2.88	83.42%	6.39%
n-212	n-212-13	*Pseudomonas fluorescens*	690	11,774	59.84%	7.76	16.46%	8.86%
n-212	n-212-2	*Rahnella aquatilis*	214	9,084	54.22%	1.93	13.76%	3.29%
p-214	p-214-25	*Roseburia intestinalis*	72	44,892	41.94%	2.59	54.01%	10.74%
p-207	p-207-24	*Roseburia intestinalis*	111	29,792	43.57%	2.55	39.47%	10.14%
n-210	n-210-39	*Roseburia intestinalis*	160	30,427	42.40%	3.44	55.96%	5.97%

p-502	p-502-12	*Roseburia intestinalis*	116	42,854	42.41%	3.50	75.67%	5.79%
n-213	n-213-24	*Roseburia intestinalis*	153	15,156	43.69%	1.88	24.43%	4.42%
p-500	p-500-3	*Roseburia intestinalis*	196	12,999	43.18%	2.31	53.22%	3.60%
n-216	n-216-25	*Rothia mucilaginosa*	162	11,291	59.45%	1.73	53.04%	5.19%
n-212	n-212-32	*Ruminococcus albus*	90	47,776	42.98%	2.72	91.91%	10.79%
n-210	n-210-47	*Ruminococcus albus*	82	62,510	42.78%	2.66	96.76%	4.82%
p-208	p-208-24	*Ruminococcus bromii*	104	22,243	40.74%	1.84	63.97%	9.78%
p-219	p-219-7	*Ruminococcus gnavus*	90	35,713	43.84%	2.13	54.85%	9.14%
p-215	p-215-7	*Ruminococcus gnavus*	173	14,218	43.04%	2.14	28.42%	5.92%
n-217	n-217-13	*Ruminococcus gnavus*	60	69,382	43.57%	2.42	91.52%	4.32%
p-500	p-500-1	*Ruminococcus gnavus*	162	10,400	42.23%	1.73	59.10%	0.94%
p-502	p-502-42	*Ruminococcus lactaris*	96	17,249	43.70%	1.52	41.54%	3.18%
p-214	p-214-20	*Ruminococcus obeum*	180	15,264	43.51%	2.34	30.40%	5.92%
n-210	n-210-53	*Ruminococcus obeum*	185	12,831	41.63%	2.11	24.40%	0.99%
n-210	n-210-27	*Ruminococcus obeum*	166	10,468	44.43%	1.66	38.85%	0.85%
p-202	p-202-36	*Ruminococcus sp.*	110	34,879	41.76%	2.76	58.32%	10.09%
n-216	n-216-10	*Ruminococcus sp.*	172	11,176	42.41%	1.74	59.85%	8.96%
n-211	n-211-23	*Ruminococcus sp.*	133	19,828	41.45%	2.03	54.22%	8.55%
p-215	p-215-13	*Ruminococcus sp.*	291	13,385	40.77%	3.49	37.01%	7.96%
p-204	p-204-41	*Ruminococcus sp.*	183	25,190	41.44%	3.31	83.87%	7.88%
n-213	n-213-23	*Ruminococcus sp.*	227	9,821	42.02%	2.14	55.32%	6.75%
n-427	n-427-8	*Ruminococcus sp.*	171	26,228	41.29%	3.36	98.02%	6.02%
n-217	n-217-14	*Ruminococcus sp.*	149	19,841	42.16%	2.41	23.42%	1.44%
p-215	p-215-15	*Ruminococcus torques*	83	58,547	40.21%	2.95	65.99%	10.51%
n-216	n-216-11	*Ruminococcus torques*	67	45,580	40.95%	2.37	71.47%	9.26%
p-219	p-219-6	*Ruminococcus torques*	168	14,339	41.18%	2.09	65.28%	8.36%
n-210	n-210-50	*Ruminococcus torques*	130	26,363	41.86%	2.49	95.89%	5.68%
n-217	n-217-12	*Ruminococcus torques*	64	59,605	41.14%	2.47	94.90%	5.17%
n-210	n-210-32	*Ruminococcus torques*	138	29,662	40.22%	2.75	67.67%	4.95%
p-204	p-204-29	*Ruminococcus torques*	137	16,040	41.77%	1.86	35.33%	2.15%
p-214	p-214-7	*Serratia marcescens*	127	63,265	57.38%	5.22	52.34%	10.82%
p-207	p-207-7	*Serratia marcescens*	223	17,604	48.78%	3.43	22.82%	9.06%
n-212	n-212-11	*Serratia marcescens*	126	28,337	60.05%	2.70	14.71%	2.75%
n-212	n-212-4	*Serratia marcescens*	88	22,575	56.89%	1.56	25.93%	1.68%
p-207	p-207-1	*Serratia marcescens*	160	11,797	59.64%	1.68	3.84%	1.20%
n-503	n-503-4	*Serratia marcescens*	122	37,747	58.92%	3.43	21.48%	0.80%
p-500	p-500-18	*Serratia marcescens*	301	13,256	58.15%	3.55	30.19%	0.58%
p-502	p-502-18	*Serratia marcescens*	371	11,005	57.48%	3.70	14.86%	0.00%
n-213	n-213-17	*Streptococcus bovis*	71	27,824	37.63%	1.63	73.02%	10.80%
p-218	p-218-6	*Streptococcus bovis*	100	24,408	37.11%	1.95	50.60%	9.91%
n-423	n-423-25	*Streptococcus bovis*	76	32,594	36.97%	1.96	73.55%	9.50%
n-211	n-211-18	*Streptococcus bovis*	56	40,000	37.41%	1.64	60.53%	9.13%
p-204	p-204-23	*Streptococcus bovis*	73	37,947	37.32%	1.70	93.22%	6.69%
p-215	p-215-9	*Streptococcus bovis*	100	20,804	37.59%	1.54	66.19%	6.09%
p-200	p-200-33	*Streptococcus bovis*	66	33,891	37.33%	1.62	55.06%	3.69%
n-217	n-217-16	*Streptococcus bovis*	52	45,174	37.54%	1.66	71.81%	3.69%
p-500	p-500-20	*Streptococcus bovis*	79	34,641	37.09%	1.91	62.72%	3.06%
n-210	n-210-16	*Streptococcus bovis*	113	15,354	37.93%	1.56	48.44%	1.88%
n-212	n-212-10	*Subdoligranulum variabile*	138	22,149	59.07%	2.48	66.27%	10.80%
p-208	p-208-13	*Subdoligranulum variabile*	180	13,711	56.24%	2.08	85.27%	9.91%
n-210	n-210-14	*Subdoligranulum variabile*	217	12,841	55.83%	2.50	89.99%	5.01%
p-502	p-502-21	*Subdoligranulum variabile*	92	25,702	60.40%	1.80	62.21%	3.46%
n-216	n-216-6	*Sutterella wadsworthensis*	94	23,339	49.66%	1.85	68.60%	9.24%
p-202	p-202-8	*Sutterella wadsworthensis*	61	75,458	55.06%	2.82	94.37%	9.24%
n-211	n-211-4	*Sutterella wadsworthensis*	101	31,114	55.89%	2.36	78.62%	5.67%
n-210	n-210-31	*Sutterella wadsworthensis*	28	115,568	49.51%	2.14	94.58%	4.20%
n-427	n-427-21	*Sutterella wadsworthensis*	49	75,276	49.39%	2.26	97.70%	4.20%
p-200	p-200-34	*Sutterella wadsworthensis*	83	36,635	62.67%	2.32	98.06%	3.57%
n-217	n-217-4	*Sutterella wadsworthensis*	88	46,787	55.20%	2.72	98.11%	2.84%
p-214	p-214-4	*Unknown*	83	84,998	54.84%	4.63	55.57%	10.99%
p-208	p-208-26	*Unknown*	16	210,367	36.62%	1.63	59.95%	10.85%
p-202	p-202-3	*Unknown*	46	57,997	48.81%	2.32	63.94%	10.69%
p-215	p-215-39	*Unknown*	100	20,802	28.47%	1.60	37.38%	10.44%

n-211	n-211-9	*Unknown*	31	119,654	26.65%	1.55	93.59%	10.33%
p-208	p-208-12	*Unknown*	54	60,174	47.60%	2.45	54.79%	10.14%
n-213	n-213-9	*Unknown*	54	61,390	33.60%	1.96	83.48%	9.96%
n-212	n-212-34	*Unknown*	106	41,324	41.63%	3.19	88.80%	9.91%
p-206	p-206-9	*Unknown*	25	159,217	28.57%	2.75	79.82%	9.79%
n-212	n-212-39	*Unknown*	112	28,678	42.22%	2.42	42.34%	9.26%
n-212	n-212-30	*Unknown*	137	19,018	45.40%	2.01	40.83%	9.21%
p-206	p-206-20	*Unknown*	63	74,746	36.75%	3.61	97.72%	9.09%
p-204	p-204-14	*Unknown*	135	18,382	46.98%	1.92	40.96%	9.07%
n-211	n-211-41	*Unknown*	126	19,291	46.84%	2.03	46.00%	9.07%
p-207	p-207-9	*Unknown*	79	34,789	47.91%	2.17	46.82%	8.78%
p-204	p-204-8	*Unknown*	38	70,998	46.15%	2.01	93.59%	8.59%
p-202	p-202-32	*Unknown*	170	13,598	42.59%	2.05	38.15%	8.53%
p-214	p-214-29	*Unknown*	228	33,700	52.66%	4.88	41.20%	8.49%
n-212	n-212-15	*Unknown*	121	16,199	30.00%	1.66	85.56%	8.47%
n-212	n-212-3	*Unknown*	136	32,019	55.83%	3.26	36.35%	8.36%
n-212	n-212-28	*Unknown*	93	36,272	45.25%	2.23	59.24%	8.18%
p-218	p-218-26	*Unknown*	135	16,615	41.00%	1.80	34.41%	7.77%
p-502	p-502-6	*Unknown*	216	20,512	29.65%	3.41	49.28%	7.50%
n-209	n-209-23	*Unknown*	48	97,307	41.45%	2.82	98.24%	7.02%
p-208	p-208-41	*Unknown*	76	33,278	40.53%	2.01	43.18%	7.01%
n-212	n-212-27	*Unknown*	108	21,086	45.21%	1.87	39.65%	6.76%
n-210	n-210-68	*Unknown*	99	36,966	41.37%	2.27	69.67%	6.72%
p-202	p-202-34	*Unknown*	134	17,853	43.91%	1.88	36.65%	6.58%
n-211	n-211-14	*Unknown*	167	12,109	34.42%	1.81	48.26%	6.51%
n-209	n-209-1	*Unknown*	126	61,383	50.92%	4.69	89.73%	6.39%
n-503	n-503-17	*Unknown*	87	39,842	48.66%	2.68	70.22%	6.13%
n-209	n-209-31	*Unknown*	22	174,431	42.29%	1.88	93.80%	6.10%
p-502	p-502-24	*Unknown*	134	22,264	49.11%	2.25	46.00%	6.09%
n-213	n-213-29	*Unknown*	145	17,937	50.07%	2.05	36.03%	5.98%
p-206	p-206-7	*Unknown*	151	12,747	28.61%	1.71	39.81%	5.97%
n-209	n-209-24	*Unknown*	73	56,454	40.85%	2.44	92.63%	5.95%
n-209	n-209-22	*Unknown*	128	30,904	38.25%	2.83	82.51%	5.93%
n-427	n-427-18	*Unknown*	106	36,638	39.76%	2.71	89.35%	5.89%
n-210	n-210-48	*Unknown*	71	62,773	38.00%	2.73	98.65%	5.77%
n-210	n-210-35	*Unknown*	65	55,605	33.22%	2.30	93.45%	5.64%
p-204	p-204-13	*Unknown*	143	12,557	48.16%	1.62	41.65%	5.55%
n-210	n-210-67	*Unknown*	105	19,896	34.21%	1.85	88.56%	5.47%
n-209	n-209-16	*Unknown*	121	28,638	45.15%	2.46	57.18%	5.41%
p-502	p-502-13	*Unknown*	138	28,606	43.98%	2.78	67.52%	5.40%
n-209	n-209-21	*Unknown*	81	44,088	36.21%	2.34	57.49%	5.26%
n-210	n-210-71	*Unknown*	41	63,956	37.46%	1.83	96.80%	5.23%
p-218	p-218-4	*Unknown*	92	22,579	30.09%	1.59	43.84%	5.13%
n-503	n-503-22	*Unknown*	35	93,732	40.41%	2.25	55.51%	5.04%
n-209	n-209-18	*Unknown*	84	41,571	43.97%	2.48	59.05%	5.04%
n-210	n-210-49	*Unknown*	159	27,571	59.46%	3.12	75.12%	5.01%
n-217	n-217-20	*Unknown*	127	14,257	37.40%	1.51	80.47%	4.96%
n-209	n-209-25	*Unknown*	107	31,347	41.76%	2.31	80.40%	4.94%
n-423	n-423-17	*Unknown*	137	25,967	46.84%	2.81	98.47%	4.92%
n-211	n-211-39	*Unknown*	157	14,200	48.09%	1.96	38.25%	4.91%
p-502	p-502-22	*Unknown*	245	37,074	47.08%	6.15	60.07%	4.82%
n-210	n-210-65	*Unknown*	158	18,048	47.44%	2.33	46.79%	4.81%
n-503	n-503-31	*Unknown*	102	19,883	38.31%	1.67	87.04%	4.67%
p-200	p-200-37	*Unknown*	150	12,145	49.32%	1.61	54.10%	4.63%
n-209	n-209-10	*Unknown*	72	74,397	31.29%	3.36	83.20%	4.63%
n-210	n-210-36	*Unknown*	147	20,751	46.42%	2.39	44.61%	4.59%
n-503	n-503-30	*Unknown*	163	21,472	43.56%	2.73	56.25%	4.59%
n-503	n-503-43	*Unknown*	171	42,629	53.35%	4.72	34.26%	4.52%
n-210	n-210-22	*Unknown*	70	55,263	44.54%	2.61	91.93%	4.51%
p-200	p-200-27	*Unknown*	80	25,876	38.58%	1.80	79.12%	4.49%
n-209	n-209-19	*Unknown*	149	13,659	48.35%	1.83	32.15%	4.41%
n-209	n-209-17	*Unknown*	62	124,682	46.45%	4.83	52.45%	4.41%
n-503	n-503-12	*Unknown*	155	13,126	34.49%	1.83	83.71%	4.16%

p-500	p-500-10	*Unknown*	133	21,577	47.69%	2.37	61.16%	4.01%
p-200	p-200-1	*Unknown*	93	37,273	34.28%	2.48	55.45%	4.01%
n-211	n-211-37	*Unknown*	151	11,008	51.33%	1.51	26.41%	3.89%
p-204	p-204-35	*Unknown*	206	7,985	28.10%	1.64	27.47%	3.82%
n-210	n-210-64	*Unknown*	154	17,508	44.08%	2.25	34.56%	3.78%
p-500	p-500-31	*Unknown*	82	41,587	48.94%	2.29	54.48%	3.78%
n-209	n-209-34	*Unknown*	35	67,098	32.64%	1.80	86.28%	3.75%
n-209	n-209-12	*Unknown*	121	23,995	26.56%	2.22	27.44%	3.69%
n-210	n-210-24	*Unknown*	161	14,634	32.10%	2.04	38.97%	3.65%
n-423	n-423-9	*Unknown*	31	137,346	28.88%	2.70	88.94%	3.60%
n-423	n-423-15	*Unknown*	147	28,203	47.57%	2.82	62.56%	3.56%
n-503	n-503-42	*Unknown*	303	22,942	46.38%	5.25	49.19%	3.50%
p-214	p-214-18	*Unknown*	212	23,548	48.19%	3.80	30.23%	3.41%
p-502	p-502-25	*Unknown*	165	12,530	50.01%	1.88	35.75%	3.31%
n-209	n-209-33	*Unknown*	91	24,838	34.73%	1.57	50.97%	3.20%
n-423	n-423-1	*Unknown*	241	11,759	41.76%	2.54	35.02%	3.14%
n-423	n-423-11	*Unknown*	46	67,911	55.79%	2.48	94.18%	2.84%
n-209	n-209-13	*Unknown*	107	17,484	44.49%	1.51	39.61%	2.80%
n-503	n-503-29	*Unknown*	183	12,465	45.35%	2.00	20.57%	2.71%
p-200	p-200-26	*Unknown*	109	15,447	41.17%	1.54	22.54%	2.69%
p-202	p-202-26	*Unknown*	77	34,034	50.92%	2.01	76.22%	2.69%
n-209	n-209-36	*Unknown*	152	13,603	38.29%	1.91	33.05%	2.39%
n-210	n-210-55	*Unknown*	194	10,737	43.05%	1.91	24.69%	2.25%
n-503	n-503-19	*Unknown*	362	10,927	50.69%	3.73	23.47%	2.15%
n-212	n-212-1	*Unknown*	216	9,983	52.13%	2.08	13.95%	2.09%
n-210	n-210-52	*Unknown*	115	24,764	45.16%	2.17	47.45%	2.02%
n-209	n-209-29	*Unknown*	100	21,322	43.90%	1.70	30.18%	1.93%
n-209	n-209-14	*Unknown*	87	26,971	42.67%	1.79	54.34%	1.88%
n-210	n-210-56	*Unknown*	167	8,978	43.47%	1.50	26.25%	1.79%
p-502	p-502-8	*Unknown*	157	10,693	36.54%	1.68	56.48%	1.73%
n-210	n-210-43	*Unknown*	145	14,283	50.62%	1.72	52.72%	1.70%
n-210	n-210-69	*Unknown*	118	24,527	47.77%	2.20	54.22%	1.62%
p-200	p-200-38	*Unknown*	149	15,088	49.23%	1.95	41.91%	1.57%
p-502	p-502-33	*Unknown*	157	13,765	42.38%	1.85	24.17%	1.44%
n-423	n-423-18	*Unknown*	159	11,149	45.50%	1.64	25.67%	1.22%
n-423	n-423-22	*Unknown*	154	14,581	43.82%	1.92	48.21%	0.96%
n-503	n-503-14	*Unknown*	41	68,137	35.90%	1.77	43.93%	0.80%
n-210	n-210-17	*Unknown*	197	12,373	48.71%	2.24	30.24%	0.76%
n-423	n-423-31	*Unknown*	192	16,356	38.01%	2.52	5.24%	0.45%
p-202	p-202-2	*Unknown*	1	6,589	50.98%	6.59	0.00%	0.00%
p-207	p-207-13	*Veillonella parvula*	154	11,328	38.81%	1.58	22.64%	7.05%
p-502	p-502-16	*Veillonella parvula*	59	48,722	38.70%	2.06	84.25%	5.63%
n-427	n-427-17	*Veillonella parvula*	32	122,133	38.75%	2.07	98.87%	5.23%
p-200	p-200-29	*Veillonella parvula*	68	40,070	38.90%	1.83	56.84%	3.87%
n-503	n-503-34	*Veillonella ratti*	39	84,868	42.27%	2.08	96.56%	5.67%
