# Supplementary material for: Patient-Specific Bacteroides Genome Variants in Pouchitis
Source: mBio. 2016 Nov 15;7(6):e01713-16. doi: 10.1128/mBio.01713-16 (PMC5111406; doi:10.1128/mBio.01713-16)
Supplement: Table S5 — Functions of high- and low-coverage regions of p214 B. fragilis genome within HMPDACC samples. The table is supplemental to Fig. S6 and details the functions identified within the rainbow-highlighted regions of the figure. The number recorded in the first column “bin” corresponds with the number adjacent to the highlighted region of the figure. The rows adjacent to each bin include the RAST assigned function, the identity of the contig containing the function, start and stop position within the contig, and orientation of the sequence (f for forward, r for reverse). Supplemental tables are available at doi:10.6084/m9.figshare.3851478. Supplemental figures are available at doi:10.6084/m9.figshare.3851481 [file mbo005163055st5.pdf]

Table S5. Functions of high and low coverage regions of p214 B. fragilis genome within HMPDACC samples

bin	function	contig	start	stop	direction
6	FIG00411354: hypothetical protein	3	309	517	r
6	FIG00408583: hypothetical protein	3	840	1052	f
6	FIG00896355: hypothetical protein	3	905	1282	f
6	FIG00899057: hypothetical protein	3	1302	1715	f
6	FIG00938992: hypothetical protein	3	3071	3325	f
6	FIG00937084: hypothetical protein	3	3348	3575	f
6	FIG00897394: hypothetical protein	3	3506	3817	f
6	FIG00403252: hypothetical protein	3	3853	4176	f
6	FIG00938196: hypothetical protein	3	4297	4515	f
6	Lysozyme-related protein	3	4573	5097	r
6	Conjugative transposon protein TraQ	3	5121	5659	r
6	Conjugative transposon protein TraP @ DNA primase (EC 2.7.7.-)	3	5603	6630	r
6	Conjugative transposon protein TraO	3	6506	7078	r
6	Conjugative transposon protein TraN	3	7084	8067	r
6	Conjugative transposon protein TraM	3	8143	9444	r
6	Mobile element protein	3	9434	9751	r
6	Conjugative transposon protein TraJ	3	10848	11390	r
6	Conjugative transposon protein TraI	3	11425	12051	r
6	Conjugative transposon protein TraH	3	12078	12422	r
6	Conjugative transposon protein TraG	3	12497	12979	r
6	Tyrosine type site-specific recombinase	3	13407	14710	f
9	Tyrosine type site-specific recombinase	3	13407	14710	f
9	clindamycin resistance transfer factor BtgB	3	18109	18630	r
9	clindamycin resistance transfer factor BtgA	3	19064	19279	r
6	Conjugative transposon protein TraG	3	23504	25408	r
6	Conjugative transposon protein TraD	3	26312	27037	r
6	Conjugative transposon protein TraC	3	27057	27407	r
6	Conjugative transposon protein TraB	3	27413	27853	r
6	Conjugative transposon protein TraA	3	27856	28959	r
6	hypothetical protein clusted with conjugative transposons, BF0131	3	29324	29749	f
6	Putative mobilization protein BF0133	3	31009	33009	f
6	Putative acetyltransferase / Putative acetyltransferase, GNAT family / DNA-3-methyladenine glycosylase (EC 3.2.2.20) / GMP synthase [glutamine-hydrolyzing] (EC 6.3.5.2)	3	33070	34209	r
6	tetracycline resistance element mobilization regulatory protein RteC	3	34304	34942	r
6	FIG00937326: hypothetical protein	3	35137	35556	r
6	tetracycline resistance element regulator RteB - Bacteroides thetaiotaomicron	3	36352	37014	r
6	FIG00939874: hypothetical protein	3	37157	39460	r
6	Tetracycline resistance protein TetQ	3	39667	40929	r
6	putative DNA methylase	3	42057	47894	r
6	FIG00938731: hypothetical protein	3	46842	48017	f
6	FIG00936811: hypothetical protein	3	47887	48473	r
6	DNA topoisomerase III, Bacteroidales-type (EC 5.99.1.2)	3	48476	50543	r
6	FIG00937566: hypothetical protein	3	50655	52214	r
6	FIG00938987: hypothetical protein	3	52238	52585	r
6	FIG00936922: hypothetical protein	3	52625	53005	r
6	FIG00938698: hypothetical protein	3	52749	53444	f
6	FIG00407580: hypothetical protein	3	53419	53781	f

6	FIG00417797: hypothetical protein	3	53868	54227	r
6	Mobile element protein	3	54243	55475	r
8	Replication protein	5	228	791	r
4	Xaa-Pro aminopeptidase (EC 3.4.11.9)	13	240760	242529	r
4	SSU ribosomal protein S21p	13	242648	242890	f
4	Integrase, site-specific recombinase	13	242943	243842	f
4	Ribosome hibernation protein YhbH	13	243863	244159	f
4	Translation elongation factor Tu	13	244758	245909	f
4	Preprotein translocase subunit SecE (TC 3.A.5.1.1)	13	246068	246238	f
4	Transcription antitermination protein NusG	13	246261	246800	f
4	LSU ribosomal protein L11p (L12e)	13	246864	247304	f
4	LSU ribosomal protein L1p (L10Ae)	13	247323	248015	f
4	LSU ribosomal protein L10p (P0)	13	248037	248540	f
4	LSU ribosomal protein L7/L12 (P1/P2)	13	248598	248933	f
4	DNA-directed RNA polymerase beta subunit (EC 2.7.7.6)	13	249077	252886	f
4	DNA-directed RNA polymerase beta' subunit (EC 2.7.7.6)	13	253027	257316	f
4	Integral membrane protein	13	257398	258531	r
4	Transglycosylase-associated protein	13	259362	259604	f
4	FIG00649583: hypothetical protein	13	260244	260552	f
4	SSU ribosomal protein S12p (S23e)	13	260803	261171	f
4	SSU ribosomal protein S7p (S5e)	13	261312	261836	f
4	Translation elongation factor G	13	261893	263968	f
4	SSU ribosomal protein S10p (S20e)	13	264095	264394	f
4	LSU ribosomal protein L3p (L3e)	13	264416	265030	f
4	LSU ribosomal protein L4p (L1e)	13	265036	265641	f
4	LSU ribosomal protein L23p (L23Ae)	13	265676	265963	f
4	LSU ribosomal protein L2p (L8e)	13	265972	266787	f
4	SSU ribosomal protein S19p (S15e)	13	266814	267083	f
4	LSU ribosomal protein L22p (L17e)	13	267122	267529	f
4	SSU ribosomal protein S3p (S3e)	13	267544	268269	f
4	LSU ribosomal protein L16p (L10e)	13	268296	268727	f
4	LSU ribosomal protein L29p (L35e)	13	268736	268930	f
4	SSU ribosomal protein S17p (S11e)	13	268930	269178	f
4	LSU ribosomal protein L14p (L23e)	13	269202	269558	f
4	LSU ribosomal protein L24p (L26e)	13	269588	269902	f
4	LSU ribosomal protein L5p (L11e)	13	269908	270462	f
4	SSU ribosomal protein S14p (S29e) @ SSU ribosomal protein S14p (S29e), zinc-independent	13	270472	270756	f
4	SSU ribosomal protein S8p (S15Ae)	13	270826	271218	f
4	LSU ribosomal protein L6p (L9e)	13	271234	271803	f
4	LSU ribosomal protein L18p (L5e)	13	271828	272169	f
4	SSU ribosomal protein S5p (S2e)	13	272178	272693	f
4	LSU ribosomal protein L30p (L7e)	13	272659	272880	f
4	LSU ribosomal protein L15p (L27Ae)	13	272899	273357	f
4	Preprotein translocase secY subunit (TC 3.A.5.1.1)	13	273365	274708	f
4	Methionine aminopeptidase (EC 3.4.11.18)	13	274726	275520	f
4	Translation initiation factor 1	13	275530	275736	f
4	SSU ribosomal protein S13p (S18e)	13	275904	276281	f
4	SSU ribosomal protein S11p (S14e)	13	276308	276679	f
4	SSU ribosomal protein S4p (S9e)	13	276811	277407	f
4	DNA-directed RNA polymerase alpha subunit (EC 2.7.7.6)	13	277422	278405	f
4	LSU ribosomal protein L17p	13	278421	278888	f
4	FIG00404648: hypothetical protein	13	279033	279446	f
4	FIG00404196: hypothetical protein	13	279492	280094	f

8	FIG00407386: hypothetical protein	19	168	953	r
8	FIG00407441: hypothetical protein	19	1452	1646	r
8	Addiction module toxin, Txe/YoeB	19	1660	1929	r
8	FIG00413440: hypothetical protein	19	1932	2174	r
8	repA	19	2593	3615	f
8	FIG00405109: hypothetical protein	19	3661	3885	r
7	putative outer membrane protein	20	186	821	r
7	FIG00408725: hypothetical protein	20	830	1483	r
7	FIG00411591: hypothetical protein	20	2003	2776	r
7	Conjugative transposon protein TraN	20	3158	3994	r
7	Conjugative transposon protein TraM	20	3999	5105	r
7	FIG00761799: membrane protein	20	5115	5540	r
7	Conjugative transposon protein TraK	20	5543	6136	r
7	Conjugative transposon protein TraJ	20	6238	7209	r
7	FIG00404931: hypothetical protein	20	7205	7852	r
7	Conjugative transposon protein TraG	20	8549	11386	r
7	Conjugative transposon protein TraE	20	11696	12007	r
7	FIG00761799: membrane protein	20	12030	12371	r
7	DNA primase TraC	20	13535	14539	f
1	hypothetical protein	23	187294	187599	f
1	FIG00404705: hypothetical protein	23	188135	188338	f
1	Single-stranded DNA-binding protein	23	189095	189460	f
1	FIG00405798: hypothetical protein	23	189955	190479	f
1	FIG00410669: hypothetical protein	23	190947	191258	f
1	FIG00411713: hypothetical protein	23	191836	192105	f
1	conserved hypothetical protein related to phage	23	192671	192922	f
1	serine type site-specific recombinase	23	194447	195133	r
1	FIG00896953: hypothetical protein	23	195324	196370	f
1	FIG00404129: hypothetical protein	23	196386	196835	f
1	FIG00897673: hypothetical protein	23	196858	197238	f
1	FIG00899205: hypothetical protein	23	197241	197795	f
1	FIG00898120: hypothetical protein	23	198833	200344	f
1	putative outer membrane protein	23	200401	201840	f
1	hypothetical protein	23	204075	204557	f
1	putative DNA primase	23	207062	208738	r
1	putative hemagglutinin	23	209630	211183	r
1	putative DNA primase	23	211198	213420	r
1	FIG00896847: hypothetical protein	23	213469	215256	r
1	putative cell wall endopeptidase family protein	23	215575	216406	r
1	FIG00897171: hypothetical protein	23	216442	217083	r
1	FIG00899176: hypothetical protein	23	217099	217782	r
1	FIG00897775: hypothetical protein	23	217801	218490	r
1	FIG00408532: hypothetical protein	23	218474	218854	r
1	FIG00899363: hypothetical protein	23	218972	220660	r
1	FIG00402755: hypothetical protein	23	220668	222233	r
1	FIG00897411: hypothetical protein	23	222262	222483	r
1	FIG00899284: hypothetical protein	23	222507	223262	r
1	Membrane proteins related to metalloendopeptidases	23	223706	224062	f
1	FIG00898496: hypothetical protein	23	224179	225012	r
1	putative ribose phosphate pyrophosphokinase	23	226009	226566	f
1	Dihydrofolate reductase (EC 1.5.1.3)	23	227424	227933	r
1	conserved hypothetical phage-like protein	23	230572	231156	r
1	putative DNA methylase	23	231666	236624	f
3	FIG00936918: hypothetical protein	31	5999	8341	r

3	FIG00937600: hypothetical protein	31	8344	9282	r
3	FIG00939378: hypothetical protein	31	11409	12284	r
3	FIG00937346: hypothetical protein	31	12301	13212	r
3	ATP-dependent Clp protease ATP-binding subunit ClpA	31	13221	15656	r
3	FIG00938542: hypothetical protein	31	15656	17428	r
3	FIG01101450: hypothetical protein	31	18506	19882	f
3	Single-stranded DNA-binding protein	31	23337	23747	f
3	FIG00403362: hypothetical protein	31	24753	24977	f
3	archaeal ATPase, fused to C-terminal DUF234 domain	31	25081	26472	r
3	FIG00407633: hypothetical protein	31	26673	26900	r
3	FIG00407633: hypothetical protein	31	28996	29149	r
3	FIG00405292: hypothetical protein	31	29350	29472	f
3	FIG00402868: hypothetical protein	31	29548	29688	f
3	Putative mobilization protein BF0133	31	30275	32458	r
3	FIG00403811: hypothetical protein	31	33216	33869	r
3	FIG00407458: hypothetical protein	31	33894	34400	r
3	Conjugative transposon protein TraN	31	34406	35245	r
3	Conjugative transposon protein TraM	31	36258	37427	r
3	FIG00403140: hypothetical protein	31	37965	38258	r
3	Conjugative transposon protein TraK	31	38295	38906	r
3	FIG01290742: hypothetical protein	31	39623	40465	r
3	FIG00896865: hypothetical protein	31	40483	41250	r
3	Adenine-specific methyltransferase (EC 2.1.1.72)	31	41322	42086	r
3	FIG00897793: hypothetical protein	31	42303	42986	r
3	FIG00898215: hypothetical protein	31	43025	45553	r
3	FIG00897908: hypothetical protein	31	45561	46196	r
3	Conjugative transposon protein TraG	31	46199	48907	r
3	FIG00897980: hypothetical protein	31	49021	49311	r
3	Conjugative transposon protein TraE	31	49326	49700	r
3	FIG00898505: hypothetical protein	31	49765	50148	r
3	FIG00896660: hypothetical protein	31	50090	50656	r
3	DNA primase (EC 2.7.7.-)	31	50777	51661	r
3	FIG00403804: hypothetical protein	31	51771	52937	r
3	putative excisionase	31	52855	53250	r
3	tyrosine type site-specific recombinase	31	54337	55464	r
3	no significant homology.	31	57777	58772	r
3	NifU-related domain containing protein	31	58796	59494	r
10	L-alanyl-gamma-D-glutamyl-L-diamino acid endopeptidase	36	535	663	f
10	Retron-type RNA-directed DNA polymerase (EC 2.7.7.49)	36	692	1190	f
1	FIG00410894: hypothetical protein	41	365	808	f
1	FIG00408654: hypothetical protein	41	811	1287	f
1	FIG00404086: hypothetical protein	41	1312	1569	f
1	Zeta toxin	41	1559	1924	f
1	zinc metalloproteinase Mpr protein	41	1941	2624	r
1	conserved hypothetical protein	41	2940	3329	f
1	conserved hypothetical protein	41	3884	5597	r
1	hypothetical protein	41	6627	7019	r
1	FIG00897050: hypothetical protein	41	7045	8599	r
1	Resolvase	41	8681	9316	r
1	Putative DNA-binding protein in cluster with Type I restriction-modification system	41	9339	10160	r
1	p5482_32	41	10179	10493	r
1	FIG00403362: hypothetical protein	41	11547	11798	f
1	Zeta toxin	41	11935	12435	f

1	Cell wall endopeptidase, family M23/M37	41	12497	13129	r
1	FIG00405317: hypothetical protein	41	13140	13916	f
1	FIG00408957: hypothetical protein	41	13921	15477	f
1	FIG00404083: hypothetical protein	41	15485	16591	f
1	DNA topoisomerase III domain	41	17783	18616	f
1	Integration host factor alpha subunit	41	18981	19256	f
1	Putative mobilization protein BF0133	41	20388	22331	f
1	putative phage related transcriptional regulator	41	22423	22710	f
1	Conjugative transposon protein TraA	41	23500	24120	r
1	putative mobilization protein	41	24139	25752	r
1	Integrase	43	5469	6647	r
7	putative DNA-binding protein	47	133	366	f
7	FIG00408941: hypothetical protein	47	706	1086	f
7	FIG00413905: hypothetical protein	47	1436	1744	f
7	Phage replication protein	47	2640	3782	f
